# Wnt7a is a novel inducer of β-catenin-independent tumor-suppressive cellular senescence in lung cancer

**DOI:** 10.1038/onc.2015.2

**Published:** 2015-03-02

**Authors:** R K Bikkavilli, S Avasarala, M Van Scoyk, J Arcaroli, C Brzezinski, W Zhang, M G Edwards, M K K Rathinam, T Zhou, J Tauler, S Borowicz, Y A Lussier, B A Parr, C D Cool, R A Winn

**Affiliations:** 1 Department of Pulmonary, Critical Care, Sleep and Allergy, College of Medicine Research Building, University of Illinois at Chicago, Chicago, IL, USA; 2 Division of Medical Oncology, School of Medicine, University of Colorado Anschutz Medical Campus, Aurora, CO, USA; 3 Department of Pediatrics, College of Medicine, University of Illinois at Chicago, Chicago, IL, USA; 4 Division of Pulmonary Sciences and Critical Care Medicine, School of Medicine, University of Colorado Anschutz Medical Campus, Aurora, CO, USA; 5 Department of Hematology and Oncology, College of Medicine, University of Illinois at Chicago, Chicago, IL, USA; 6 University of Arizona Cancer Center, Tucson, AZ, USA; 7 Center for functional genomics, University of Albany, Rensselaer, NY, USA; 8 Department of Pathology, School of Medicine, University of Colorado Anschutz Medical Campus, Aurora, CO, USA; 9 Jesse Brown Veterans Affairs Medical Center, Chicago, IL, USA

## Abstract

Cellular senescence is an initial barrier for carcinogenesis. However, the signaling mechanisms that trigger cellular senescence are incompletely understood, particularly *in vivo*. Here we identify Wnt7a as a novel upstream inducer of cellular senescence. In two different mouse strains (C57Bl/6J and FVB/NJ), we show that the loss of Wnt7a is a major contributing factor for increased lung tumorigenesis owing to reduced cellular senescence, and not reduced apoptosis, or autophagy. Wnt7a-null mice under *de novo* conditions and in both the strains display E-cadherin-to-N-cadherin switch, reduced expression of cellular senescence markers and reduced expression of senescence-associated secretory phenotype, indicating a genetic predisposition of these mice to increased carcinogen-induced lung tumorigenesis. Interestingly, Wnt7a induced an alternate senescence pathway, which was independent of β-catenin, and distinct from that of classical oncogene-induced senescence mediated by the well-known p16^INK4a^ and p19^ARF^ pathways. Mechanistically, Wnt7a induced cellular senescence via inactivation of S-phase kinase-associated protein 2, an important alternate regulator of cellular senescence. Additionally, we identified Iloprost, a prostacyclin analog, which initiates downstream signaling cascades similar to that of Wnt7a, as a novel inducer of cellular senescence, presenting potential future clinical translational strategies. Thus pro-senescence therapies using either Wnt7a or its mimic, Iloprost, might represent a new class of therapeutic treatments for lung cancer.

## Introduction

Lung cancer remains the leading cause of cancer-related deaths in the world for both men and women. Oncogenic stimuli trigger the cells to escape from the normal constraints of cell growth, adopt some, or all of the hallmarks of cancer,^1^ and undergo cellular transformation and carcinogenesis. However, in response to oncogenic stress cells activate protective mechanisms such as cellular senescence, which acts as a barrier to cellular transformation and tumor progression.^2, 3, 4, 5^ Cellular senescence is a process of permanent cell cycle arrest, which halts proliferating cells in the G1 phase of the cell cycle.^6^ Senescent cells are generally characterized by a distinct and recognizable flattened and enlarged morphology with prominent nuclei and the accumulation of vacuoles.^6^ Currently, there is no single marker attributed to senescent cells. Research over the past years has been successful in identifying and characterizing several important characteristics of a senescent cell, for example, increased senescence-associated β-galactosidase (SA-β-gal) activity,^7^ activation of the retinoblastoma protein (hypophosphorylated-Rb) as a result of increased expression of cyclin-dependent kinase (CDK) inhibitors (p16^INK4A^, p19^ARF^ and p27^KIP1^),^6, 8^ and the induction of a senescence-associated secretory phenotype (SASP).^8, 9, 10^ These responses allow the cells to remain static and unresponsive to mitogenic signals, and therefore protect the cells from repeated oncogenic insults, and tumorigenesis. Although elegant, the mechanisms driving tumor-protective cellular senescence remains incompletely understood.

Wnts are secreted glycoproteins that bind to the members of the seven transmembrane family of proteins called Frizzleds (Fzds).^11, 12, 13^ Wnts binding to Fzds initiates a complex network of events, which are either dependent on β-catenin (canonical pathway)^11, 14^ or independent of β-catenin (non-canonical pathways).^15, 16, 17, 18, 19^ The oncogenic potential of the Wnt pathway has been well established in many cancers.^20, 21^ Thus the components of Wnt/β-catenin pathway are either mutated or de-regulated in many human cancers and are known to be causal for tumorigenesis.^20, 21, 22^

Interestingly, unlike other Wnts, Wnt7a has been shown to have an important tumor-protective role, particularly in the lung.^18, 19, 23^ Strikingly, Wnt7a is lost in many non-small cell lung cancer (NSCLC) cell lines and tissues, primarily via promoter methylation^24^ and restoration of Wnt7a expression in NSCLC cell lines prompts cellular differentiation and reversal of the transformed phenotype.^18, 19^ Given the importance of Wnt7a in tumor suppression, we sought to define the mechanism/s through which Wnt7a exerts its tumor-protective role.

Using genetic studies (loss-of-function) in mice combined with whole transcriptome sequencing (RNA sequencing (RNA-seq)), we define Wnt7a as a novel inducer of cellular senescence. We show for the first time that the increased lung tumorigenesis observed in Wnt7a-null mice is directly due to reduced cellular senescence and SASP. We also show that the Wnt7a-mediated cellular senescence operate as an alternate senescence pathway, distinct from that of the well-known and well-studied oncogene-induced senescence (OIS). Thus compounds that can induce Wnt7a expression, or function similarly to Wnt7a, would have a great potential for pro-senescence therapy in lung cancer. Of note, Iloprost, a prostacyclin analog and small-molecule Wnt7a mimic, also induces cellular senescence. Thus Wnt7a appears to act as a master regulator of cellular senescence, and Wnt7a-mediated tumor-suppressive cellular senescence may represent a new class of future therapeutic treatments of lung cancer.

## Results

### Loss of Wnt7a leads to increased lung tumorigenesis in an *in vivo* lung cancer model

**Figure 1:**
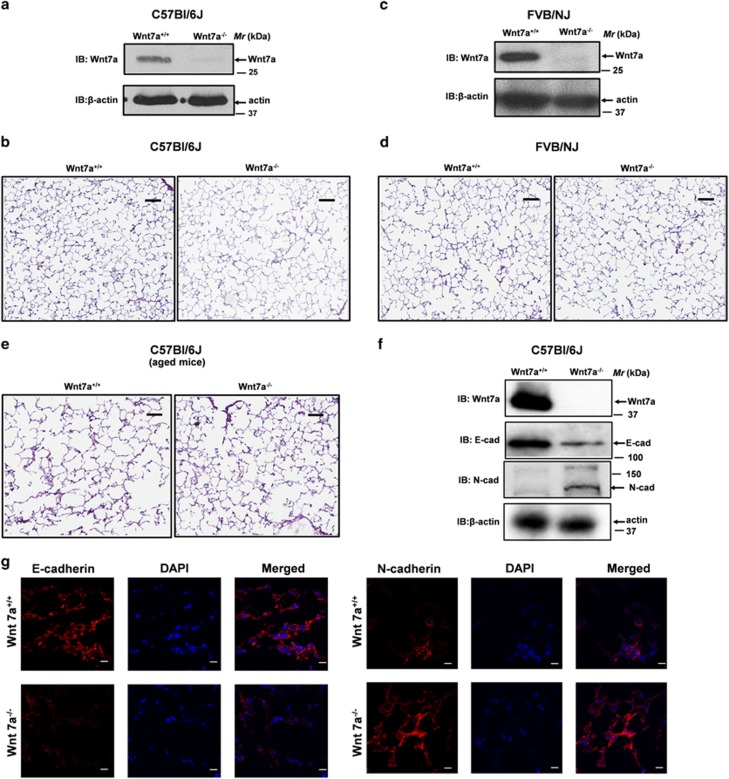

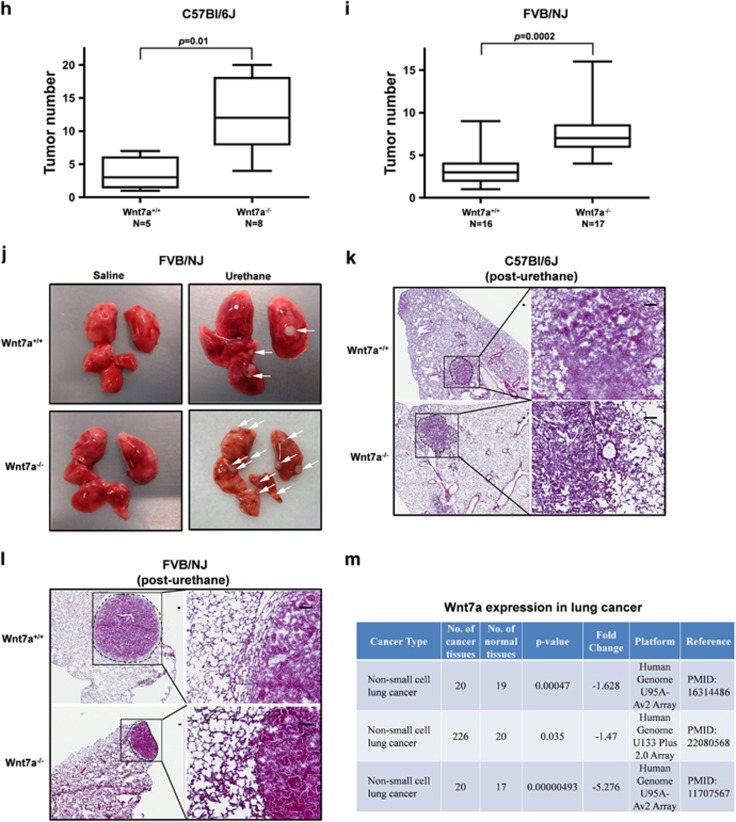
Loss of Wnt7a leads to decrease in lung epithelial cell markers and increased lung tumorigenesis. Representative images of the western blotting analysis of Wnt7a expression in lung lysates derived from wild-type and Wnt7a-null mice either in C57Bl/6J (**a**), or FVB/NJ (**c**) strains. (**b**, **d**, **e**) Representative images of the histological sections of the lungs of the indicated genotypes that were stained with hematoxylin and eosin (H&E) stain displaying no gross differences in the lung architectures among Wnt7a-null mice and wild-type littermate controls. Scale bar: 100 μm. (**f**) Representative western blotting images of the lung lysates of wild-type (*n*=3) and Wnt7a-null mice (*n*=3) displaying reduced expression of epithelial cell marker (E-cadherin) and increased expression of mesenchymal cell marker (N-cadherin) in the Wnt7a-null mice. (**g**) Histological sections of the lungs were fixed and stained with either E-cadherin or N-cadherin antibodies, and the expression of E-cadherin and N-cadherin were visualized by indirect immunofluorescence and confocal microscopy. Representative images of Wnt7a-null mice and wild-type littermate controls are displayed in the figure. Scale bar: 10 μm. (**h**, **i**) Wild-type and Wnt7a-null mice in C57Bl/6J (**h**) or FVB/NJ (**i**) strains were given urethane as described in the Materials and methods section. The mice were later euthanized and dissected to assess lung tumorigenesis. Box plots display the number of lung tumors developed in the wild-type and Wnt7a-null mice in response to urethane. Statistical significance was determined using Mann–Whitney non-parametric test. (**j**) Representative images of the lung tumors from wild-type and Wnt7a-null mice. (**k**, **l**) Representative images of the histological sections of the lung tissues with tumors stained with H&E as detailed in the Materials and methods section. The edges of the tumors were identified with a broken line. Although wild-type mice developed adenomas with soft borders, majority of Wnt7a-null mice developed adenocarcinomas, as evidenced by spiculated borders. Scale bar: 100 μm. (**m**) Wnt7a expression was lost in the majority of NSCLCs as determined from the Oncomine database search.

Previous studies have highlighted Wnt7a as a tumor suppressor in a number of epithelial-cell-derived cancers.^18, 19, 23^ In the current study, we utilized the Wnt7a germline null mouse as a physiologically relevant model to evaluate the role of Wnt7a in lung tumorigenesis^25^ (The Jackson laboratory, Bar Harbor, ME, USA; stock number 004715). For these studies, we bred the Wnt7a-null mice until they were congenic in both C57Bl/6J and FVB/NJ strains. The absence of Wnt7a in these mice was confirmed via immunoblots with Wnt7a-specific antibodies (Figures 1a–c). The Wnt7a-null mice are known to have mild limb deformities and sterility in both homozygous males and females.^25^ Interestingly, evaluation of the lung architecture of Wnt7a-null mice showed no gross morphological or histological differences from their wild-type littermates (Figures 1b–d). Moreover, spontaneous lung tumors were not observed in either the young (3 months, Figures 1b–d) or aged (18 months, Figure 1e) Wnt7a-null mice. However, the lungs of the Wnt7a-null mice did show reduced expression of the epithelial cell marker (E-cadherin) and elevated expression of mesenchymal cell marker (N-cadherin) as determined by immunoblotting (Figure 1f) and by indirect immunofluorescence (Figure 1g), suggesting that Wnt7a expression is required for normal epithelial gene expression in the adult lung. These findings suggest that loss of Wnt7a expression is an important predisposing factor for the development of lung cancer.

To test this idea, we probed for the effects of urethane (ethyl carbamate)-induced lung tumor formation in wild type and Wnt7a-null mice, a prototypical model to study lung tumorigenesis.^26, 27, 28^ Urethane is a chemical carcinogen, which causes activating mutations in K-Ras,^27, 28^ leading to the formation of lung tumors in mice. Several studies have highlighted a strain-specific response to urethane; for example, FVB/NJ strains are susceptible to lung tumorigenesis, whereas C57Bl/6J, in contrast, are more resistant.^26, 27, 28^ Therefore, in our studies, wild type and Wnt7a null in FVB/NJ mice received the standard single dose of 1 mg/g body weight of urethane, whereas wild type and Wnt7a null in C57Bl/6J mice received weekly injections of 1 mg/g body weight of urethane for 6 weeks. The mice were euthanized and dissected after 20 weeks (FVB/NJ strains) or 40 weeks (C57Bl/6J strains) to assess the formation of lung tumors.

We observed that the Wnt7a-null mice developed more tumors (>2-fold) in both the C57Bl/6J (*N*=8), and FVB/NJ (*N*=17) strains, in comparison to wild-type littermate controls (Figures 1h–j). Furthermore, significant differences were also observed upon histological examination of the lungs of wild-type and Wnt7a-null mice after urethane exposure. Although wild-type mice developed adenomas (Figures 1k and l), majority of Wnt7a-null mice, in contrast, developed adenocarcinomas, as illustrated by spiculated borders^29, 30^ (Figures 1k and l). Taken together, these data suggest that Wnt7a has an important tumor-suppressive role in lung carcinogenesis.

In strong support of our hypothesis, analysis of public microarray data sets for Wnt7a expression revealed that: (1) Wnt7a is predominantly expressed in epithelial cells (GEO profiles, GDS1402/NM_004625.1_PROBE1/WNT7A), and (2) there is a profound loss of Wnt7a expression in several human lung cancer studies (Figure 1m). Considering that lung cancers are predominantly epithelial cell-derived, our data highlights the importance of Wnt7a expression in the lung epithelium and its loss as a major contributing factor for carcinogenesis.

### The tumor-suppressor role of Wnt7a is independent of β-catenin signaling

**Figure 2:**
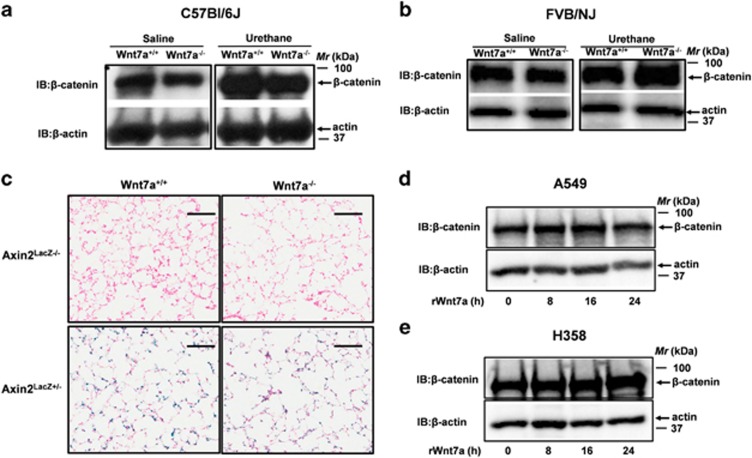
Tumor-suppressive effects of Wnt7a are independent of β-catenin signaling. Representative images of the western blotting analysis of β-catenin expression in the lung lysates derived from wild-type (*n*=5) and Wnt7a-null mice (*n*=5) in C57Bl/6J (**a**) or FVB/NJ (**b**) strains treated with either saline or urethane. No apparent differences in β-catenin expression were observed among wild-type and Wnt7a-null mice. (**c**) Representative images of the histological sections of mouse lungs derived from Wnt7a^+/+^/Axin2^Lacz+/−^ (*n*=3) and Wnt7a^−/−^/Axin2^Lacz+/−^ (*n*=3) mice stained for the expression of *β-galactosidase* gene as described in the Materials and methods section. *β-Galactosidase* expression was similar in the lungs of wild-type and Wnt7a-null mice carrying Axin2^Lacz+/−^ allele. Human lung adenocarcinoma cells (A549, **d**) and human bronchioalveolar carcinoma cell (H358, **e**) that are devoid of Wnt7a expression were stimulated with recombinant Wnt7a for the indicated periods of time. The cells were later lysed, and β-catenin expression was determined by probing the blots with anti-β-catenin antibodies. Representative blots from three independent highly reproducible experiments are displayed in the figure. Scale bar: 100 μm.

β-catenin is a well-established proto-oncogene, and mutations of this gene are common in many cancers.^20, 21^ Moreover, Wnts are known regulators of β-catenin signaling.^11, 12, 13^ To determine whether β-catenin signaling also had an important role in Wnt7a-mediated lung tumorigenesis, we probed the expression levels of β-catenin in the lungs of wild-type and Wnt7a-null mice (Figure 2). For these studies, total lung lysates of wild-type and Wnt7a-null mice were subjected to immunoblotting with anti-β-catenin-specific antibodies (Figures 2a and b). When examined before and after urethane treatment, the expression of β-catenin was similar in Wnt7a-null mice and their wild-type littermates (Figures 2a and b). Additionally, we tested the effects of Wnt7a on the expression of Axin2, a well-characterized target of Wnt/β-catenin signaling that provides an accurate read-out of Wnt/β-catenin signaling activity *in vivo*.^31^ We bred Wnt7a heterozygotes (Wnt7a^+/−^) to Axin2^LacZ^ reporter mice in which one copy of the Axin2 locus was replaced with a *β-galactosidase* gene. Consistent with the effects of Wnt7a on β-catenin expression, lung tissue sections of Wnt7a^+/+^/Axin2^Lacz+/−^ and Wnt7a^−/−^/Axin2^Lacz+/−^ mice showed similar expression of *β-galactosidase* gene as determined by β-galactosidase assays (Figure 2c). These data strongly suggest that the tumor-suppressive effects of Wnt7a are not influenced by β-catenin expression and/or β-catenin signaling activity. Of note, stimulation of cultured NSCLC cell lines (that is, A549 and H358) that are devoid of Wnt7a^18^ with recombinant Wnt7a also failed to affect β-catenin expression (Figures 2d and e). Therefore, by using several distinct approaches, we showed that the tumor-suppressive effects of Wnt7a are independent of β-catenin.

### Gene expression analysis in Wnt7a-null mice identified several hallmarks of cancer

**Figure 3:**
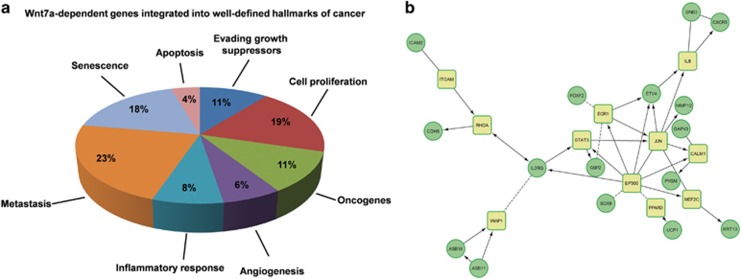
Lung-specific gene expression in wild-type and Wnt7a-null mice. (**a**) Total RNA isolated from urethane-treated wild-type (*n*=3) and Wnt7a-null mice (*n*=3) in C57Bl/6J strain were subjected to transcriptome analysis using RNA-seq. Genes that showed a false discovery rate of <0.05, log2 fold-change of >1.3 and the number of mapped reads >10.4 were considered to be significant. The gene list is manually categorized by gene function integrated into the well-defined hallmarks of cancer using NCBI resources (for example, UniGene, Pubmed and so on). The percentage of genes in each category to the total number of genes identified are shown in the pie chart. (**b**) Gene network analysis for the identified senescence-associated genes using Cytoscape plug-in from the Reactome predicted an inherent co-ordination among the identified genes. The senescence-associated genes are identified in green circles, while the reference genes/proteins in the Cytoscape are identified in yellow squares.

In order to identify the mechanism/s through which Wnt7a has a tumor-suppressive role, we performed high-throughput RNA-seq on the lung tissues of wild-type and Wnt7a-null mice after urethane treatment (*n*=3). The RNA-seq analysis revealed strong upregulation of cell proliferation-related genes (19%), oncogenes (11%) and metastasis-related genes (23%) in the Wnt7a-null mice (Figure 3a, see RNA-seq gene list in Supplementary Files). Although oncogenic insults would result in the induction of host defense mechanisms such as cell death (apoptosis or autophagy) or cellular senescence, it was striking that 18% of the genes affected by the loss of Wnt7a were associated with cellular senescence (Figure 3a, Supplementary Table S1), whereas only 4% of the genes affected by Wnt7a loss were associated with apoptosis, and very few with autophagy (Figure 3a). Moreover, genes required to induce cellular senescence were downregulated in the Wnt7a-null mice, while the genes that block cellular senescence, in strong contrast, were upregulated (Supplementary Table). Furthermore, gene network analysis using Cytoscape (http://www.cytoscape.org/) plug-in from the Reactome (http://www.reactome.org/) also revealed that the identified genes (green circles) were highly connected, indicating that there might be an inherent co-ordination among the identified genes either through similar biological function/s and/or correlated expression (Figure 3b). Additionally, several genes that are known to regulate epithelial–mesenchymal transition (EMT) were also observed to be de-regulated in the Wnt7a-null mice (Supplementary Table S2). Based on these data, we hypothesize that the failure of the Wnt7a-null mice to undergo cellular senescence is the critical predisposing factor for increased carcinogen-induced lung tumorigenesis.

### Wnt7a-null mice display reduced cellular senescence

**Figure 4:**
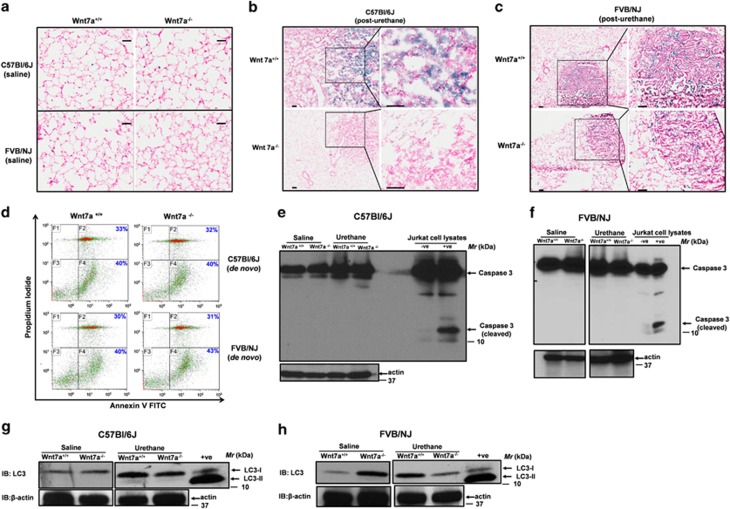
Wnt7a-null mice display reduced cellular senescence. (**a**) Histological sections of the lungs of the indicated genotypes and in the indicated strains were stained for SA-β-galactosidase activity as detailed in the Materials and methods section and are displayed in the figure. The lung sections of wild-type and Wnt7a-null mice showed no differences in the basal staining for SA-β-galactosidase activity. (**b**, **c**) Lung sections of wild-type (*n*=3) and Wnt7a-null mice (*n*=3) treated with urethane were stained for SA-β-galactosidase activity as detailed in the Materials and methods section and are displayed in the figure. Lung sections of the Wnt7a-null mice showed reduced staining for SA-β-galactosidase activity in comparison to wild-type littermate controls. (**d**) Lungs of wild-type (*n*=3) and Wnt7a-null mice (*n*=3) in C57Bl/6J and FVB/NJ strains were harvested, and the cells were stained for Annexin V/propidium iodide double staining followed by flow cytometry. No differences in the percentage of Annexin V-positive cells were observed among wild-type and Wnt7a-null mice. (**e**, **f**) Representative images of the western blotting analysis for Caspase 3 activity in the lung lysates of wild-type (*n*=5) and Wnt7a-null mice (*n*=5) in C57Bl/6J (**e**) or FVB/NJ (**f**) strains treated with either saline or urethane displayed no detectable cleaved Caspase 3 expression. (**g**, **h**) Representative images of the western blotting analysis for LC3 expression in the lung lysates of wild-type (*n*=5) and Wnt7a-null mice (*n*=5) in C57Bl/6J (**g**) or FVB/NJ (**h**) strains treated with either saline or urethane displayed no detectable LC3-II band. Scale bar: 100 μm.

Based on our RNA-seq data, we characterized the Wnt7a-null mice for cellular senescence in greater detail by measuring SA-β-gal activity, a hallmark of senescent cells.^7^ Histological analysis of the lung sections of the wild-type and Wnt7a-null mice showed no difference in basal staining for SA-β-gal activity (Figure 4a). However, a large number of SA-β-gal-positive cells, predominantly in the tumor area, were observed in lung sections of the wild-type mice after urethane treatment (Figures 4b and c), whereas Wnt7a-null mice, on the contrary, showed reduced or no SA-β-gal staining (Figures 4b and c). These data suggest that Wnt7a-null mice indeed have reduced capabilities to undergo cellular senescence in the lung.

We also evaluated the effects of Wnt7a loss on other cellular tumor-suppressive mechanisms, such as apoptosis and autophagy. Annexin V/propidium iodide staining of lung cells isolated from wild-type and Wnt7a-null mice (*n*=3) showed no significant changes in the percentages of Annexin V-positive cells (F4 quadrant) between wild-type and Wnt7a-null mice in both the strains (Figure 4d). Consistent with the Annexin V staining, no differences in Caspase3 activities were observed in the lung lysates of wild-type and Wnt7a-null mice treated with either saline or urethane (Figures 4e and f). Furthermore, probing the lung lysates with LC3 antibodies, an autophagosome marker, showed only the LC3-I (18 KDa) form, not the active, autophagosome membrane-bound LC3-II (16 KDa) form. These data suggest that the tumor-suppressive effects of Wnt7a were not mediated via the induction of autophagy (Figures 4g and h). Taken together, these data strongly suggest that reduced cellular senescence, but not apoptosis, or autophagy, is a major contributor to the increased lung tumorigenesis seen in the Wnt7a-null mice.

### Wnt7a regulates alternate cellular senescence via inactivation of S-phase kinase-associated protein 2 (SKP2)

**Figure 5:**
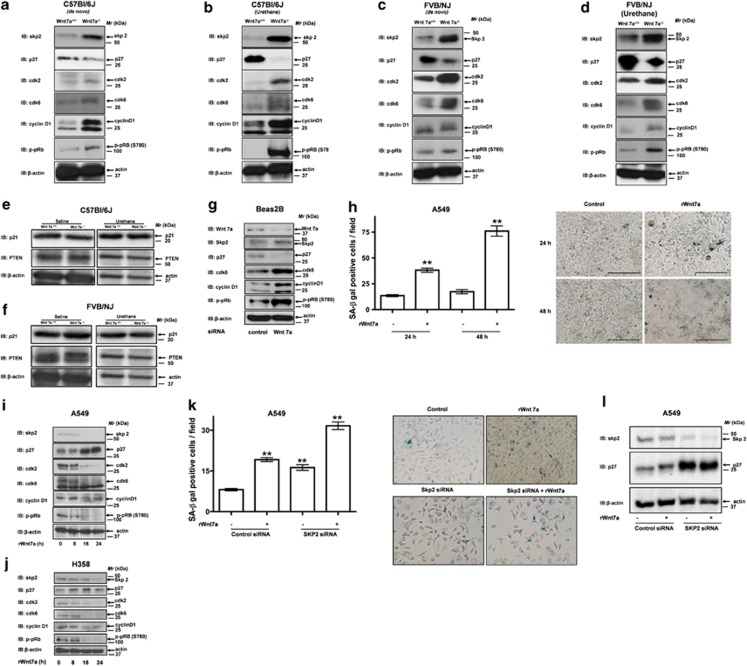
Wnt7a regulates Rb phosphorylation via inactivation of SKP2. (**a**–**f**) Representative images of the western blotting analysis for the indicated proteins in the lung lysates of wild-type (*n*=5) and Wnt7a-null mice (*n*=5) in C57Bl/6J, or FVB/NJ strains treated with either saline or urethane. (**g**) Human bronchial epithelial cells (Beas2B) were treated with either control siRNA or Wnt7a siRNA for 48 h. The cell lysates were later probed for the indicated proteins, and the representative images are displayed in the figure. (**h**–**j**) Human lung adenocarcinoma cells (A549 or H358), which are devoid of Wnt7a expression, were stimulated with recombinant Wnt7a for the indicated periods of time. The cells were later analyzed for SA-β-galactosidase activity (**h**) or the expression of indicated senescence markers (**i**, **j**) as described in the Materials and methods section. Representative blots from three independent highly reproducible experiments are displayed in the figure. For SA-β-galactosidase staining, left panel indicates the number of SA-β-gal-positive cells/field, and representative images are displayed to the right. **P<0.01; versus control. Scale bar: 200 μm. (**k**, **l**) Human lung adenocarcinoma cells (A549), which are devoid of Wnt7a expression, were treated with SKP2 siRNAs prior to the stimulation with recombinant Wnt7a for 24 h. The cells were later analyzed for SA-β-galactosidase activity (**k**) or the expression of indicated senescence markers (**l**) as described in the Materials and methods section. Representative blots from three independent highly reproducible experiments are displayed in the figure. For SA-β-galactosidase staining, left panel indicates the number of SA-β-gal-positive cells/field, and representative images are displayed to the right. **P<0.01; versus control.

CDK inhibitors (for example, p16^INK4a^/p19^ARF^/p27^kip1^) are critical for the induction of cellular senescence via blocking the phosphorylation of retinoblastoma tumor-suppressor protein (Rb).^6, 8, 32^. In order to identify the CDK inhibitor/s regulated by Wnt7a, we probed the lung lysates of wild-type and Wnt7a-null mice for p16^INK4a^, p19^ARF^ and p27^kip1^ protein levels via immunoblotting. Interestingly, compared with wild-type littermate controls, p27^kip1^ expression was reduced in both saline- and posturethane-treated Wnt7a-null mice in both the strains (Figures 5a–d, quantified blots are included in Supplementary Figures S2 and S8). Notably, p16^INK4a^ and p19^ARF^, well-known key regulators of OIS,^33, 34, 35, 36, 37^ were not regulated by Wnt7a (Supplementary Figures S13 and S14). Moreover, the expression of p21^WAF1^ and PTEN (phosphatase and tensin homolog), other known regulators of cellular senescence,^38, 39^ were also similar in the Wnt7a-null mice and their wild-type littermate controls (Figures 5e and f), suggesting that Wnt7a-induced cellular senescence is not mediated by p16^INK4a^, p19^ARF^, p21^WAF1^ or PTEN.

On the contrary, SKP2, an alternative regulator of cell cycle progression via ubiquitination-mediated degradation of p27^kip1^,^40, 41, 42, 43^ was robustly upregulated in both the saline- and urethane-treated Wnt7a-null mice (Figures 5a–d, quantified blots are included in Supplementary Figures S1 and S7). These data imply that the downregulation of p27^kip1^ in Wnt7a-null mice was due to the upregulation of SKP2 expression. Consistent with the changes in SKP2 and p27^kip1^ expression, the levels of phosphorylated (p-Rb-S780) retinoblastoma proteins in Wnt7a-null mice were dramatically increased as well (Figures 5a–d, quantified blots are included in Supplementary Figures S6 and S12). Cyclin D1, which is critical for Ser780 phosphorylation of Rb (Figures 5a–d, quantified blots are included in Supplementary Figures S5 and S11), was also upregulated in Wnt7a-null mice, along with CDK2 and CDK6, components of the CDK/cyclin complex (Figures 5a–d, quantified blots are included in Supplementary Figures S3). Similar results were obtained when using the lung lysates of wild-type and Wnt7a-null in FVB/NJ mice (Figure 5c and d, quantified blots are included in Supplementary Figures S7–S12). In sum, Wnt7a inactivates SKP2 leading to the stabilization of p27^kip1^. Stabilized p27^kip1^ subsequently blocks the phosphorylation of Rb by CDKs, activating Rb to induce cellular senescence. Furthermore, these data also suggest that Wnt7a regulates an alternate senescence pathway, which is distinct from OIS, via the SKP2/p27^kip1^-mediated regulation of Ser780 phosphorylation of Rb.

Consistent with the alterations in the expression of the alternate senescence pathway in the Wnt7a-null mice, small interference RNA (siRNA)-mediated knockdown of Wnt7a in cultured non-transformed bronchial epithelial cells also showed reduced expression of p27^kip1^ and increased Ser780 phosphorylation of Rb (Figure 5g). In order to provide a direct evidence for Wnt7a regulation of an alternative senescence pathway, we stimulated NSCLC cells (that is, A549), which were deficient in Wnt7a expression, with recombinant Wnt7a (rWnt7a, Figures 5f and g). Stimulation of cells with rWnt7a induced robust cellular senescence marked by an increase in SA-β-gal-positive cells, in a time-dependent manner (Figure 5h). Remarkably, rWnt7a treatment also induced prominent changes in the cell morphology, that is, flattening of the cells and loss in cell–cell junctions, which is typical for a senesce nt cell (Figure 5h). Moreover, rWnt7a stimulation also induced a marked decrease in SKP2 expression and a corresponding increase in p27^kip1^ expression, in a time-dependent manner (Figure 5i). Furthermore, Wnt7a stimulation also blocked the activities of CDKs as detected by the apparent reduction in Ser780 phosphorylation of Rb (Figure 5i). Similar results were obtained when H358 cells (p53-null NSCLC cell lines) were stimulated with rWnt7a (Figure 5j), suggesting that the Wnt7a-induced senescence is independent of p53 expression. In order to provide additional evidence to support our hypothesis that Wnt7a-induced senescence was mainly through modulation of SKP2, we analyzed the effects of Wnt7a-mediated senescence in SKP2 knockdown A549 cells. We observed an increase in the number of SA-β-gal-positive cells in SKP2 knockdown A549 cells (Figure 5k), an effect similar to that of Wnt7a treatment alone (Figure 5k). Additionally, knock down of SKP2 enhanced the effects of Wnt7a on cellular senescence (Figure 5k). Furthermore, SKP2 knockdown also induced marked increase in p27^kip1^ expression, both in the absence and presence of Wnt7a (Figure 5l). In sum, these findings demonstrate that Wnt7a is a novel inducer of tumor-suppressive cellular senescence via the modulation of the expression of SKP2-mediated alternate senescence pathway.

### Wnt7a regulates SASP

**Figure 6:**
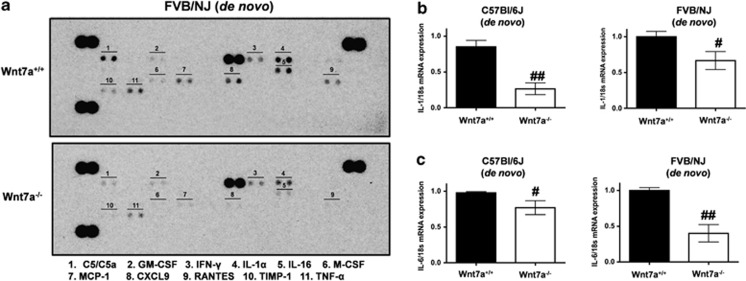
Wnt7a regulates SASP. Equal amounts of protein lysates of wild-type and Wnt7a-null mice in FVB/NJ strain were probed against a mouse cytokine array (**a**) (ARY006, R&D) to profile the relative levels of select cytokines and chemokines in wild-type and Wnt7a-null mice as per the manufacturer's recommendations. A representative image of the cytokine array identifying differentially expressed cytokines was displayed in the image. (**b**, **c**). Total RNA isolated from wild-type (*n*=3) and Wnt7a-null mice (*n*=3) were used to generate cDNAs. The cDNAs were later used to determine the expression of IL1α (**b**) and IL6 (**c**) via quantitative PCR. The expression of IL1α and IL6 were normalized to the expression of 18S ribosomal RNA and are displayed in the figure. ^##^*P*<0.01; versus Wnt7a^+/+^.

Senescent cells, in addition to withstanding oncogenic stress, can also send distress signals alarming neighboring cells of potential oncogenic threat via secreting a number of secretory factors, a feature known as the SASP.^8, 9, 10^ Interestingly, probing lung protein lysates of wild-type and Wnt7a-null mice against antibody arrays revealed several chemokines/cytokines, whose expression was decreased in the Wnt7a-null mice (Figure 6a, interleukin 1α (IL1α), IL16, macrophage colony-stimulating factor, monocyte chemotactic protein-1, C-X-C motif chemokine ligand 9, regulated on activation, normal T cell expressed and secreted (RANTES), tissue inhibitors of metalloproteinase-1, and tumor necrosis factor-α). Among the identified cytokines, IL1α was significantly reduced in the Wnt7a-null mice (Figure 6a). Expression of IL1α by the senescent cells is considered to be a critical and early manifestation of SASP.^9^ Moreover, quantitative PCR analysis of lung total RNA also revealed reduced expression of IL1α mRNA in the Wnt7a-null mice (Figure 6b), further corroborating our antibody array data (Figure 6a). As IL1α is an upstream regulator of senescence-associated IL6/IL8 network,^9^ we also analyzed the expression of IL6 in the lungs of wild-type and Wnt7a-null mice by quantitative PCR (Figure 6c). Consistent with the reduced expression of IL1α, IL6 expression was also reduced in Wnt7a-null mice (Figure 6c). In total, these results strongly indicate that Wnt7a expression was not only critical for the induction of cellular senescence but also for a SASP.

### Iloprost regulates cellular senescence

**Figure 7:**
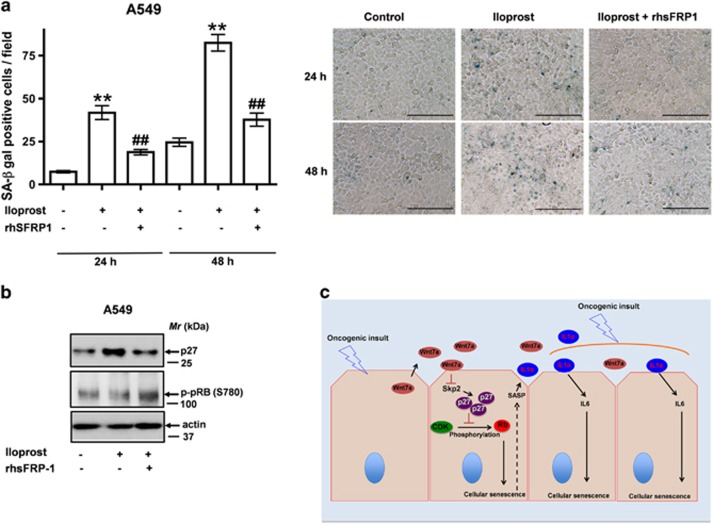
Iloprost stimulates cellular senescence. Human lung adenocarcinoma cells (A549), which are devoid of Wnt7a expression, were stimulated with Ilprost (20 μM) for the indicated periods of time either in the presence or absence of recombinant human sFRP1 (0.5 μg/ml). The cells were later analyzed for SA-β-galactosidase activity (**a**) or the expression of the indicated senescence markers (**b**) as described in the Materials and methods section. Representative blots from three independent highly reproducible experiments are displayed in the figure. For SA-β-galactosidase staining, left panel indicates the number of SA-β-gal-positive cells/field and the representative images are displayed in the right panel. **P<0.01; versus control.##*P*<0.01; versus rWnt7a. Scale bar: 200 μm. (**c**) Schematic representation of Wnt7a-mediated cellular senescence and a SASP.

Iloprost, a synthetic analog of prostacyclin (PGI_2_), was shown to reduce the tumor number in mice via receptor binding and initiation of downstream signaling cascades similar to that of Wnt7a.^44^ Moreover, Iloprost was shown to improve endobronchial dysplasia in former smokers in a recent clinical trial.^45^ As Iloprost was shown to function as a Wnt7a mimic,^44^ we tested whether Iloprost treatment of NSCLC cells could trigger cellular senescence similar to Wnt7a (Figures 5f and g). Notably, treatment of A549 cells with Iloprost also induced robust cellular senescence in a time-dependent manner, as revealed by an increase in the number of SA-β-gal-positive cells with a flattened cell morphology (Figure 7a), an effect similar to that of Wnt7a treatment (Figure 5f). Iloprost treatment also showed a strong induction of p27^kip1^ expression and a corresponding decrease in Rb phosphorylation, hallmarks of cellular senescence (Figure 7b). Moreover, Iloprost-induced cellular senescence was blocked by secreted Frizzled-related protein (sFRP1), a cysteine-rich domain containing protein homologous to Fzds (Figures 7a and b), suggesting the specificity of Iloprost for Fzd receptors. Therefore we identified a novel role for Iloprost in regulating cellular senescence and as a potential therapeutic pro-senescence therapy treatment for lung cancers.

## Discussion

For many years, cellular senescence had been considered a phenomenon only observed in cell culture systems.^46^ Although cellular senescence has been well appreciated in the aging process, in recent years several groups have highlighted the importance of cellular senescence in cancer.^2, 3, 4, 5, 37, 38^ However, the key players and their roles in driving the cells toward cellular senescence remain incompletely understood. Importantly, the signaling mechanisms that allow cells to decide whether or not to senesce remain largely unknown. In this study, we identified Wnt7a as a novel inducer of tumor-suppressive cellular senescence in the lung. The new knowledge garnered from this study offers new and exciting therapeutic opportunities, which can be tested both in culture and *in vivo*.

Our study has led to several important and novel findings. First, we found that Wnt7a mediates a novel senescence pathway, which is distinct from that of OIS. Our identification of Wnt7a as a novel inducer of cellular senescence has great therapeutic implications, as compounds that can stimulate Wnt7a expression and/or mimic its function will be able to drive cellular senescence even in the absence of DNA damage and protect from carcinogenesis.

Second, Wnt7a-mediated cellular senescence is independent of β-catenin. Although, increased expression and/or stabilizing mutations of β-catenin have been found to be pro-tumorigenic in several human cancers, particularly in colorectal cancers,^20, 21, 22^ the germline deletion of Wnt7a had no effect on β-catenin expression in the lung (Figure 2). Moreover, mutations in the components of the β-catenin-dependent pathway are not common in lung cancers.^47, 48^ Unlike other components of the Wnt pathway, the expression of Wnt7a has been seen to be reduced or lost in a significant number of lung cancers (Figure 1m).^18^ Additionally, Wnt7a has also been recently shown to be downregulated in 88% of clear cell renal carcinomas.^23^ Interestingly, the Wnt7a gene is located on chromosome 3p21, a ‘hot spot' where allelic deletions are frequently seen in many cancers.^49^ In addition to gene deletions, the Wnt7a promoter is hypermethylated, particularly in response to cigarette smoke condensate, which was also shown to be an important mechanism of Wnt7a inactivation.^24^ Taken together, we speculate that the constant exposure of respiratory epithelia to cigarette smoke may lead to increased lung tumorigenesis via inactivation of Wnt7a expression.

Third, we showed that cellular senescence is significantly reduced in the Wnt7a-null mice and serves as the major contributing factor for increased lung tumorigenesis. Detailed biochemical, cell biology and histopathology studies revealed that the loss of Wnt7a leads to reduced cellular senescence, as defined by the absence or reduced SA-β-gal-positive cells, altered expression of senescence markers, and SASP (Figures 4). In the current study, we have identified Wnt7a as a novel inducer of cellular senescence. Our identification of Wnt7a-mediated cellular senescence was quite unique, as: (1) Wnt7a-induced senescent cells displayed flattened cell morphology (Figures 5h and 7a), (2) Wnt7a-triggered cellular senescence was transduced via the activation of an alternative SKP2/p27^kip1^/p-Rb pathway, and (3) Wnt7a-mediated senescence was not mediated by p16^INK4a^, p19^ARF^ and p53/p21^WAF1^, which are important mediators of OIS. Moreover, Wnt7a-induced senescence was also observed to be independent of p53 expression (Figure 5j), indicating broader translational value of our findings. Furthermore, Wnt7a-mediated cellular senescence also was not mediated via PTEN regulation (Figures 5e and f), another known inducer of a novel type of cellular senescence.^38^

Fourth, we have identified Wnt7a as a novel regulator of SKP2. SKP2 is frequently upregulated in cancers and is an important regulator of CDK inhibitor p27^kip1^, critical for the induction of cellular senescence.^40, 42, 43^ Earlier, Wnt antagonist, WIF1, was shown to repress SKP2 expression in urinary bladder cancer cells,^50^ and Wnt5a was shown to suppress epithelial ovarian cancer via promoting cellular senescence^51^ in a β-catenin-dependent manner. As the anti-proliferative effects of Wnt7a were independent of β-catenin (Figure 2), we have identified a novel β-catenin-independent mechanism of regulation of SKP2 and cellular senescence by Wnt7a. Interestingly, the transcriptome sequencing studies performed on Wnt7a-null mice also identified forkhead box containing proteins (Supplementary Table) as probable Wnt7a effector proteins regulating the expression of SKP2 and p27^kip1^.^52^ In addition, Wnt7a regulation of cytokines (C-X-C motif chemokine receptor 5, intercellular adhesion molecule 2, and IL2rg; Figure 6a), which are important for the induction of SASP, also strengthens our hypothesis that Wnt7a is a novel regulator of cellular senescence and SASP.

Fifth, Wnt7a-null mice under *de novo* conditions display an E-cadherin-to-N-cadherin switch, reduced expression of cellular senescence markers and reduced expression of SASP, indicating a genetic predisposition of these mice to increased carcinogen-induced lung tumorigenesis. Although we have shown that the loss of Wnt7a was an important predisposing factor for reduced cellular senescence and increased lung tumorigenesis, several questions still remain to be addressed: (1) Genetic deletion of Wnt7a not only showed reduced expression of senescence markers (Figure 5) but also induced an E-cadherin-to-N-cadherin switch (Figures 1f and g). However, the role of E-cadherin-to-N-cadherin switch in the induction of cellular senescence remains largely unknown. (2) Many of the genes that are known to regulate the hallmarks of cancer were also de-regulated in the Wnt7a-null mice. However, the association of these genes to cellular senescence or *vice versa* also remains to be established and is of potential therapeutic interest. (3) We have shown that Wnt7a was a novel inducer of cellular senescence and SASP in the lungs. As Wnt7a is predominantly expressed in the epithelium, it is possible that the lung epithelium might have an important role in the induction of cellular senescence. Therefore, future studies should be focused on understanding the abilities of lung epithelial cells, namely, clara, alveolar type II and/or alveolar type I cells to induce cellular senescence. Considering the secretory nature of Wnt7a, the effects of Wnt7a on the neighboring stroma and their influence on cellular senescence also is a possibility. Additionally, Wnt7a-null mouse model is an important tool not only for understanding the mechanism of senescence *per se* but also for testing the efficacy of pro-senescence molecules.

Sixth, despite the effects of existing chemotherapy and several select agents on inducing cellular senescence,^53, 54^ the use of pro-senescence therapies as a potential treatment for cancers has not been fully adopted. In the current study, we have identified Wnt7a as a novel inducer of cellular senescence. Although the clinical utility of Wnt7a is very limited due to the unavailability of clinically suitable recombinant Wnt7a protein, our identification of Iloprost, a prostacyclin analog and a Wnt7a mimic, as a novel regulator of cellular senescence opens new opportunities for utilizing pro-senescence therapy in lung cancers.

In summary, our findings identified Wnt7a as a novel inducer of cellular senescence and SASP. Wnt7a inactivates SKP2 leading to the stabilization of p27^kip1^. Stabilized p27^kip1^ subsequently blocks the phosphorylation of Rb by CDKs, activating Rb to induce cellular senescence (Figure 7c). Additionally, senescent cells, although non-dividing, are metabolically active and can have profound affects on neighboring cells via secreted factors such as IL6, IL8 and other cytokines. Senescent cells upregulate the expression of IL1α that binds to IL1 receptor in a juxtacrine manner and activate the senescence-associated IL6/IL8 cytokine network (Figure 7c),^9^ thereby amplifying the senescence phenotype. In total, these responses (cellular senescence and SASP) may allow cells to withstand repeated oncogenic insults and prevent tumor formation. Therefore, the present study not only identified senescence as a beneficial phenomenon but also identified potential ways to induce cellular senescence clinically, via manipulation of Wnt7a.

## Materials and methods

### Animal studies

#### Ethics statement

Animal experiments were conducted in strict accordance with the recommendations in the Guide for the Care and Use of Laboratory Animals of the National Institutes of Health. The animals were housed in RC2 vivarium, Anschutz Medical Campus, University of Colorado, Aurora, CO, USA. The protocol (B-75611(06)1E) was approved by the Institutional Animal Care and Use Committee.

#### Wnt7a^−/−^ null mice

Wnt7a-null mice (Wnt7a^−/−^) and their wild-type (Wnt7a^+/+^) littermates are inbred strains on B6/129 S1genetic background (The Jackson Laboratory, stock number 004715). Parental stocks of Wnt7a^−/−^ and Wnt7a^+/+^ mice were a generous gift from Dr Brian A Parr (Director, Mouse Transgenic Facility, Center for Functional Genomics, State University of New York at Albany, Rensselaer, NY, USA). Wnt7a-null mice were bred until congenic in both C57Bl/6J and FVB/NJ strains. As the homozygous Wnt7a-null mice were sterile, we made use of heterozygotes for colony maintenance. Genetic backgrounds of the mice were determined using PCR of DNA from tail biopsies and western blotting with Wnt7a antibody.

#### Wnt7a^−/−^ / Axin2^LacZ/+^ mice

Axin2^tm1Wbm/J^ mice (The Jackson Laboratory, stock number 009120) harbor an Axin2^LacZ^ (also called conductin^LacZ^) mutation that abolishes endogenous *Axin2* gene function and expresses NLS-*lacZ* under the control of the endogenous *Axin2* promoter/enhancer regions. For generating Wnt7a^−/−^/Axin2^LacZ/+^ mice, Axin2^tm1Wbm/J^ mice were backcrossed with Wnt7a heterozygotes in C57Bl/6J strain. As Wnt7a-null mice are sterile, generating Wnt7a-null mice homozygous for Axin2^LacZ^ is extremely difficult. We therefore used wild-type and Wnt7a-null mice with one allele of Axin2^LacZ^ (Axin2^LacZ/+^) in our studies. Similar strategy was used for understanding the role of Ror2 in Wnt5A signaling.^55^ The genetic background of the mice was determined by PCR through DNA obtained from tail biopsies.

#### Urethane treatment

Six-to-eight-week-old mice were given intraperitoneal injections of either 0.9% saline or urethane (1 mg/g body weight). Wild-type and Wnt7a-null mice in FVB/NJ strain received the standard single dose of urethane, whereas the wild-type and Wnt7a-null mice in C57Bl/6J strain received weekly injections of 1 mg/g body weight of urethane for 6 weeks. The control and experimental mice were weighed weekly to observe any changes in body weight until they were euthanized. The mice were euthanized and dissected after 20 weeks (FVB/NJ strains) or 40 weeks (C57Bl/6J strains) to assess the formation of lung tumors. Lung tumors were counted and measured using digital calipers (Fisher Scientific, Waltham, MA, USA).

### Cell culture

NSCLC cell lines A549 and human non-transformed lung epithelial cell line (Beas2B) were cultured in RPMI 1640 medium (10-040-CV, Cellgro, Mediatech Inc., Manassas, VA, USA) supplemented with 10% fetal bovine serum in a humidified 5% CO_2_ incubator at 37 °C. The cell lines were cultured bi-weekly and stocks of cell lines were passaged no more than 10 times for use in experiments. Confluent cell cultures were serum starved for 2 h, followed by the stimulation with 100 ng/ ml of recombinant Wnt7a (3008-WN, R&D systems, Inc., Minneapolis, MN, USA) for the indicated periods of time. For Iloprost studies, confluent cell cultures were stimulated with 10 μM of Iloprost (78918-13-8, Cayman Chemical, Ann Arbor, MI, USA). In experiments involving shFRP1 (5396-SF, Cayman Chemical), confluent cell cultures were first incubated with 0.5 μg/μl shFRP1 for 1 h, followed by the stimulation with Iloprost.

For Wnt7a knockdown studies, Beas2B cells were seeded in a 100-mm dish (2 × 10^6^ cells), followed by incubation for 1 h at 37 ^o^C. Wnt7a siRNAs (5 nM) or SKP2 siRNAs (100 nM) diluted in 1 ml of serum-free medium were mixed with 40 μl of Hiperfect Transfection reagent (Qiagen, Valencia, CA, USA). After incubation of the siRNA complexes for 5 min at room temperature, the siRNA complexes were added drop-wise onto the cells. The cells were incubated for 48 h and analyzed for Wnt7a knockdown. The control siRNAs (all stars negative control, no. 1027280) and Wnt7a siRNAs (SI0006620) were obtained from Qiagen (Venlo, Limburg, the Netherlands). SKP2 siRNAs (sc-36499) were obtained from Santa Cruz Biotechnology (Santa Cruz, CA, USA).

### Western blotting analysis

Lung tissue protein extracts were obtained from mouse lungs homogenized in tissue protein extraction lysis buffer containing a cocktail of 1 M HEPES, 5 M NaCl, 10% Triton X-100, 1 M DTT, 0.5% EDTA, 20 mM NaVO_3_, 10 mM PMSF, 0.5 M Na-β-glycerophosphate and protease inhibitors using Qiagen TissueLyser LT (Qiagen), while NSCLC cells were lysed in a lysis buffer (MAP kinase lysis buffer), and the western blotting analysis was carried out as previously described.^18, 19^ Anti-β-actin (A3853) and anti-β-catenin (C2206) were purchased from SIGMA (Sigma-Aldrich, St Louis, MO, USA). Anti-Caspase 3 (9662S), anti-Skp2 (4313S), anti-p27^kip^ (2552S), anti-Cdk6 (3136S), anti-CyclinD1 (2922S), anti-LC3A/B (4108S) anti-p-pRB (3590S) were purchased from Cell Signaling (Cell Signaling Technology Inc., Danvers, MA, USA) and anti-Wnt7a (SC26360) was purchased from Santa Cruz Biotechnology. Densitometric analysis was performed using the ImageJ software (NIH, Bethesda, MD, USA). Band intensities were normalized to their corresponding controls and are represented in the figures.

### Hematoxylin and eosin staining

Lungs were inflated *in situ* with 50:50 (phosphate-buffered saline (PBS): Tissue-Tek O.C.T compound (M7148-4, Cardinal Health, Fisher HealthCare, Houston, TX, USA) by intratracheal intubation, flash frozen and embedded in Tissue-Tek O.C.T compound. Hematoxylin and eosin staining was performed on 6-micron sections at the Histology and Tissue Imaging Core of the University of Illinois at Chicago (UIC, Chicago, IL, USA). Stained sections of the whole lungs were later scanned using Aperio Scanscope CS and its associated Spectrum image Management and Analysis system (Leica Biosystems Inc, Buffalo Grove, IL, USA). Snapshot images were captured at different magnifications using the ImageScope software (Leica Biosystems Inc., Richmond IL, USA).

### SA-β-gal staining

SA-β-gal staining was performed on the lung tissue sections and NSCLC cells using the Senescence Detection Kit (K320-250, BioVision, Inc. Milpitas, CA, USA) according to the manufacturer's instructions. Briefly, 10-micron lung tissue sections or NSCLC cells were fixed with the fixative solution for 10 min on ice, followed by overnight incubation with the staining solution. The next day, lung sections and cells were washed with PBS. Stained lung sections were later scanned using Aperio Scanscope CS and its associated Spectrum image Management and Analysis system. The images of NSCLC cells were captured using an inverted bright field microscope (Evos, Life Technologies, Carlsbad, CA, USA) equipped with the image capture software. The number of SA-β-Gal positive cells in each filed were manually counted and are represented in the graphs. β-Galactosidase assays were performed using the β-Galactosidase Staining Kit (K-802-250, BioVision Inc.) according to the manufacturer's instructions.

### Indirect immunofluorescence

Expression of E-cadherin and N-cadherin were detected by using indirect fluorescence and confocal microscopy. Briefly, 6-micron lung tissue sections were fixed with the fixative solution for 15 min at room temperature followed by 1 h incubation with 5% goat serum. The sections were later incubated overnight with either anti-E-cadherin or N-cadherin antibodies at 4 ^o^C. The next day, lung sections were washed in PBS and incubated with anti-mouse-Cy^3^-labeled secondary antibodies for 1 h at room temperature. After extensive washes with PBS, the sections were mounted with DAPI (4,6-diamidino-2-phenylindole) containing mounting medium. Indirect immunofluorescence images were later captured with Zeiss confocal microscope (Leica Biosystems) at the confocal microscopy facility at research resources laboratory, UIC.

### Annexin V/propidium iodide staining

For measuring apoptosis, fluorescein isothiocyanate Annexin V Apoptosis Detection Kit I was used (556547, BD Pharmingen, San Jose, CA, USA). Briefly, dissected lungs from wild-type and Wnt7a-null mice were digested with 1 mg/ml Collagenase Type IV (LS004180, Worthington Biochemical Corporation, Lakewood, NJ, USA) and 100 μg/μl DNase 1 (79254, Qiagen) for 1 h at 37 °C to obtain single cells. Isolated single cells were washed three times with PBS and red blood cells were lysed with ACK lysing buffer (A10492-01, Life Technologies). Later, cells were stained with Annexin V according to the manufacturer's protocol. Flow cytometry was performed using a Beckman Coulter Gallios (Beckman Coulter, Pasadena, CA, USA) flow cytometer at the flow cytometry core facility, Anschutz Medical Campus, University of Colorado.

### RNA isolation and real-time PCR

Total RNA from lung tissues and NSCLC cells were obtained using QIAzol (Qiagen) as per the manufacturer's recommendations. For quantitative real-time PCR, 250 ng of total RNA was reverse transcribed using random primers, and PCRs were performed using the QuntiFast SYBR Green PCR Kit (204050, Qiagen) and the Bio-Rad CFX real-time PCR detection system (Hercules, CA, USA). The primers utilized in the PCR experiments were as follows: IL1: F 5′-GGAGAGCCGGGTGACAGTATC-3′, R 5′-TCAGCCGTCTCTTCTTCAGAATC-3′ and IL6: F 5′-CCACGGCCTTCCCTACTTC-3′, R 5′-TTGGGAGTGGTATCCTCTGTGA-3′.

### RNA sequencing and data analysis

After treatment with urethane, RNA was extracted from the lungs of wild-type (*N*=3) and Wnt7a-null (*N*=3) mice as described above. cDNA libraries were constructed and sequenced on a HiSeq-2000 (Illumina, San Diego, CA, USA) at the University of Colorado Genomics and Microarray core. On an average, 50 million single-end 100 bp reads were obtained from each sample. RNA-seq analysis was later performed using Cufflinks workflow. To determine the differentially expressed genes, genes that showed a false discovery rate of <0.05, log2 fold change of >1.3 and the number of mapped reads >10.4 were considered to be significant. The gene list was later manually categorized by gene function integrated into the well-defined hallmarks of cancer via NCBI resources, such as UniGene, UniProt, Pubmed and so on. The percentage of genes in each category to the total number of genes identified are shown in the pie chart. Gene network analysis for the identified senescence-associated genes were obtained using Cytoscape plug-in from the Reactome.

### Statistical analysis

To assess the statistical significance of all the quantitative data, Student's *t*-test, Mann–Whitney non-parametric tests or one-way analysis of variance with Tukey's multiple comparison were performed.
