# Supplementary material for: Wnt7a is a novel inducer of β-catenin-independent tumor-suppressive cellular senescence in lung cancer
Source: Oncogene. 2015 Mar 2;34(42):5317–28. doi: 10.1038/onc.2015.2 (PMC4558401; doi:10.1038/onc.2015.2)
Supplement: Supplementary Figures [file onc20152x1.pdf]

Supplementary Figures S1-S14: Total lung lysates of wild-type (n=5) and Wnt7a null mice (n=8) in C57Bl/6J or FVB/NJ strains treated with either saline or 1 mg/kg b.w urethane were subjected to western blot analysis using antibodies against SKP2 (S1,S7), $p27^{kip1}$ (S2,S8), Cdk2 (S3,S9), Cdk6 (S4,S10), Cyclin D1 (S5,S11), p-pRb (S6,S12), p16 (S13, and p19 (S14). Immunoreactive bands were acurately quantified using Image J software and the normalized densitometry readings were represented in the Figures. Top panel represents the normalized band intensities to their corresponding controls, while; representative images of the blots were displayed in the bottom panel. One-way ANOVA with Tukey's multiple comparison test was used to determine the statistical significance. (**, p<0.01; increased expression versus control ($Wnt7a^{+/+}$ saline), and $^{\#\#,}$ p<0.01; decreased expression versus control ($Wnt7a^{+/+}$ saline).

S1

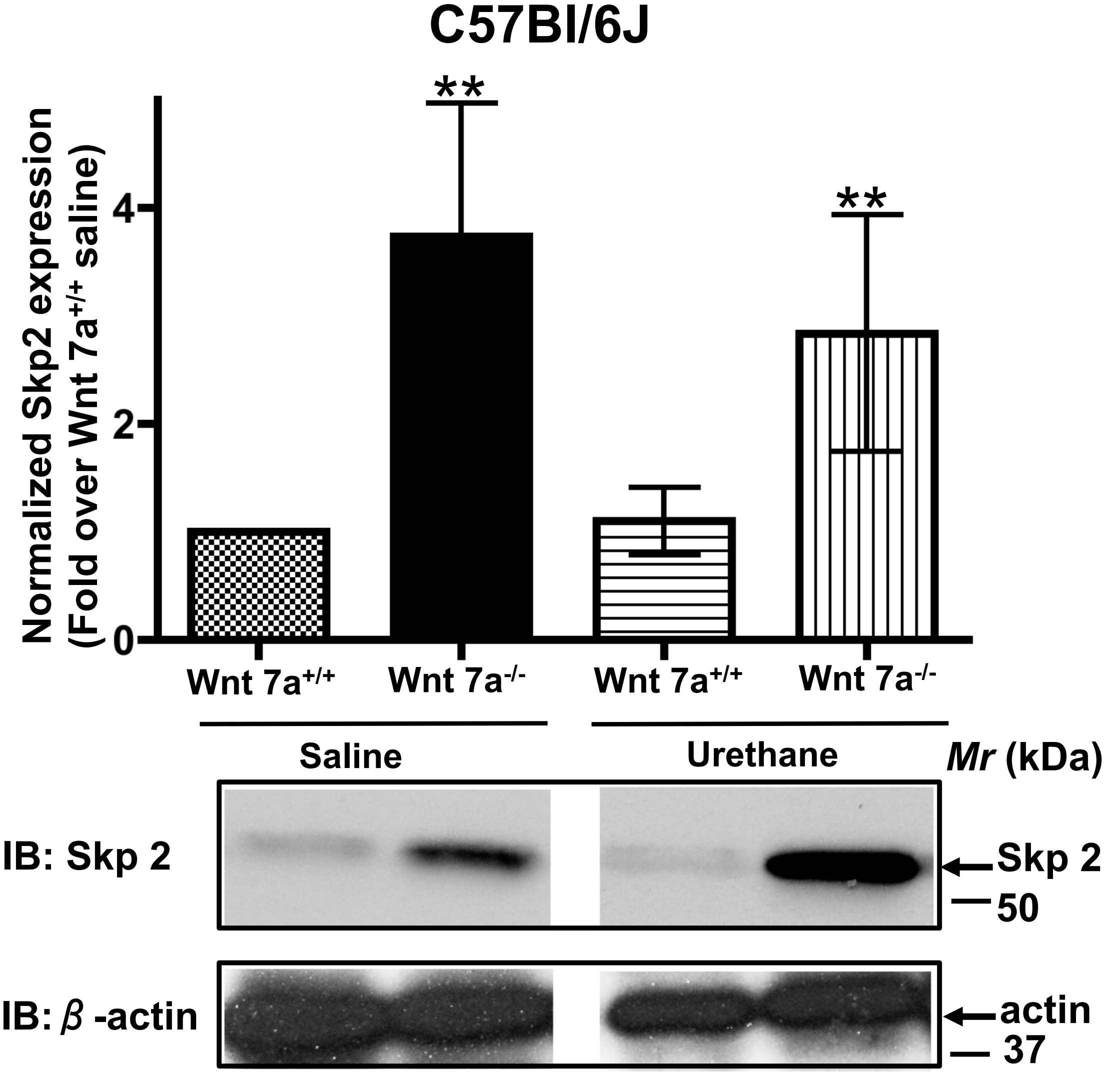
C57Bl/6J
Normalized Skp2 expression
(Fold over Wnt 7a+/+ saline)
0
2
4
**
**
Wnt 7a+/+
Wnt 7a-/-
Wnt 7a+/+
Wnt 7a-/-
Saline
Urethane
Mr (kDa)
IB: Skp 2
Skp 2
50
IB: β -actin
actin
37

S2

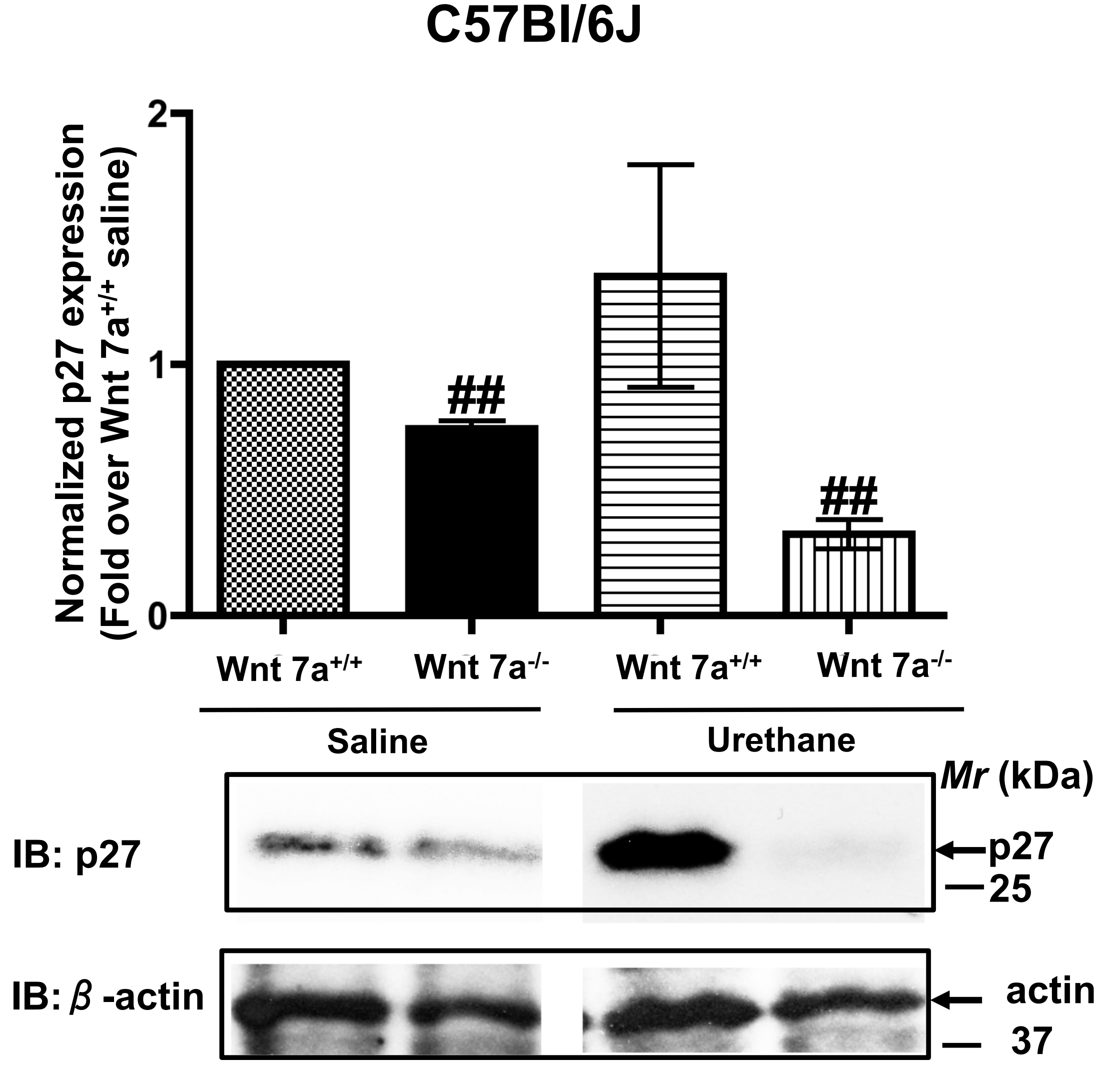
C57Bl/6J
Normalized p27 expression
(Fold over Wnt 7a+/+ saline)
2
1
0
##
##
Wnt 7a+/+
Wnt 7a-/-
Wnt 7a+/+
Wnt 7a-/-
Saline
Urethane
Mr (kDa)
IB: p27
p27
25
IB: β -actin
actin
37

S3

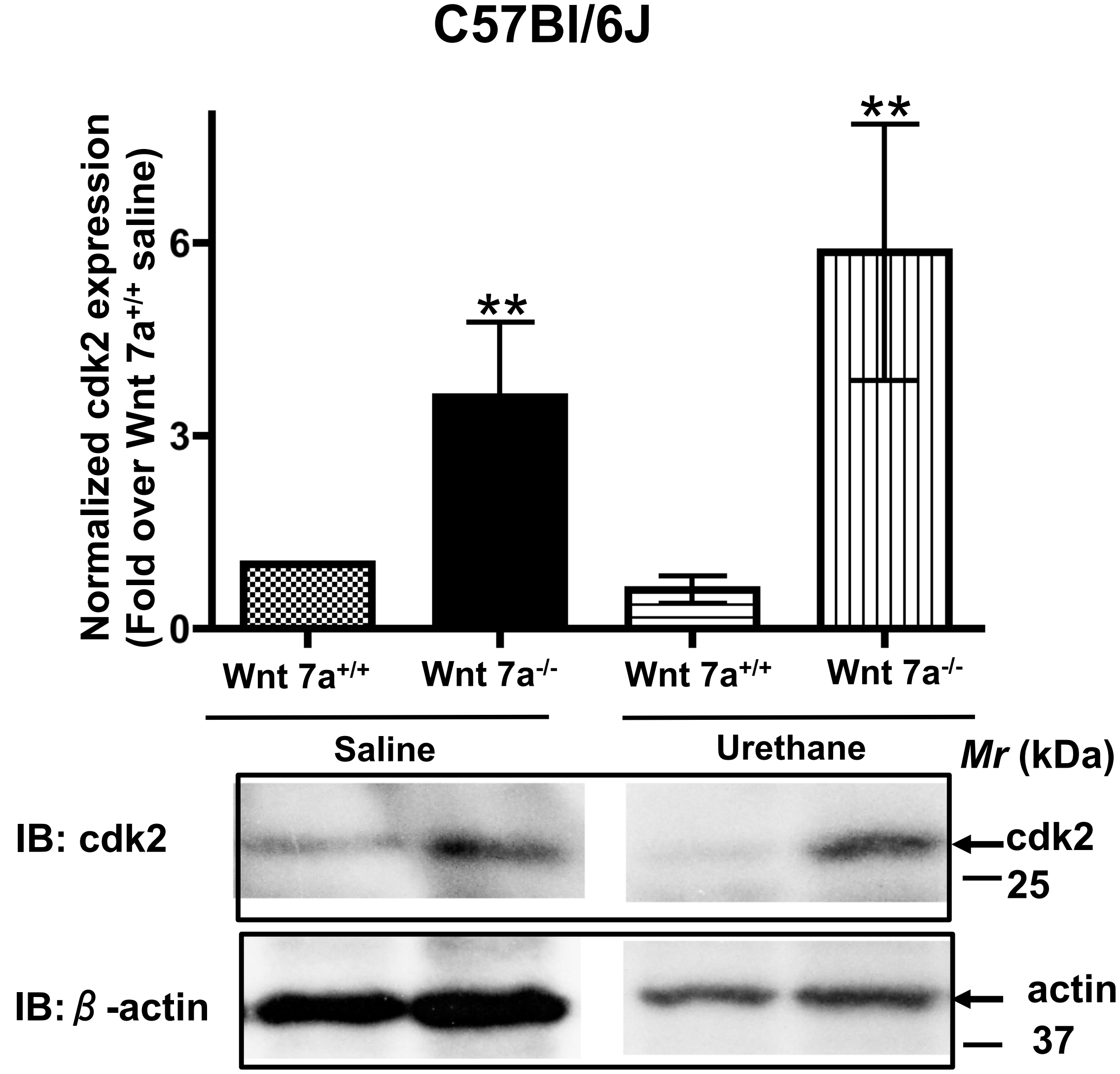
C57Bl/6J
Normalized cdk2 expression (Fold over Wnt 7a+/+ saline)
0
3
6
**
**
Wnt 7a+/+
Wnt 7a-/-
Wnt 7a+/+
Wnt 7a-/-
Saline
Urethane
Mr (kDa)
IB: cdk2
cdk2
25
IB: β -actin
actin
37

S4

C57Bl/6J

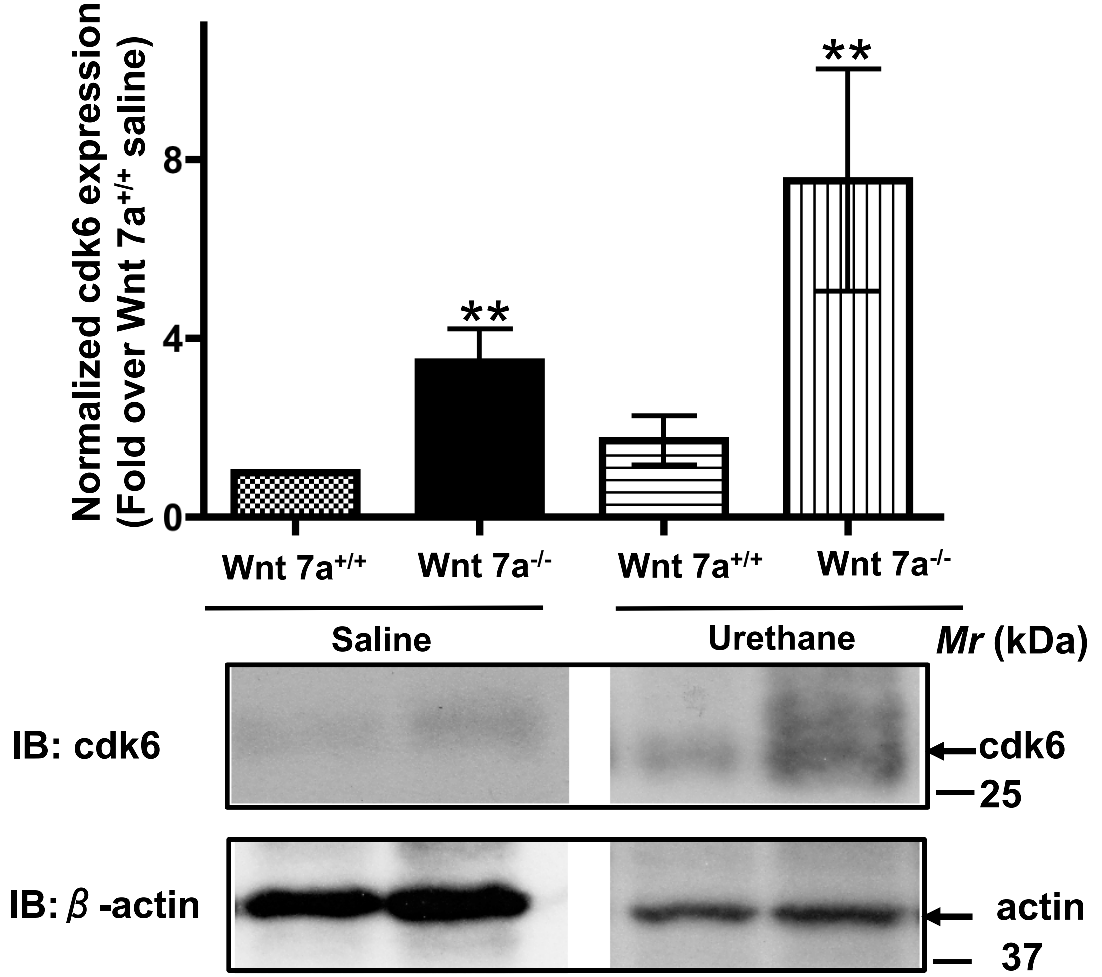

Normalized cdk6 expression
(Fold over Wnt 7a+/+ saline)
0
4
8
**
**
Wnt 7a+/+
Wnt 7a-/-
Wnt 7a+/+
Wnt 7a-/-
Saline
Urethane
Mr (kDa)
IB: cdk6
cdk6
25
IB: β -actin
actin
37

S5

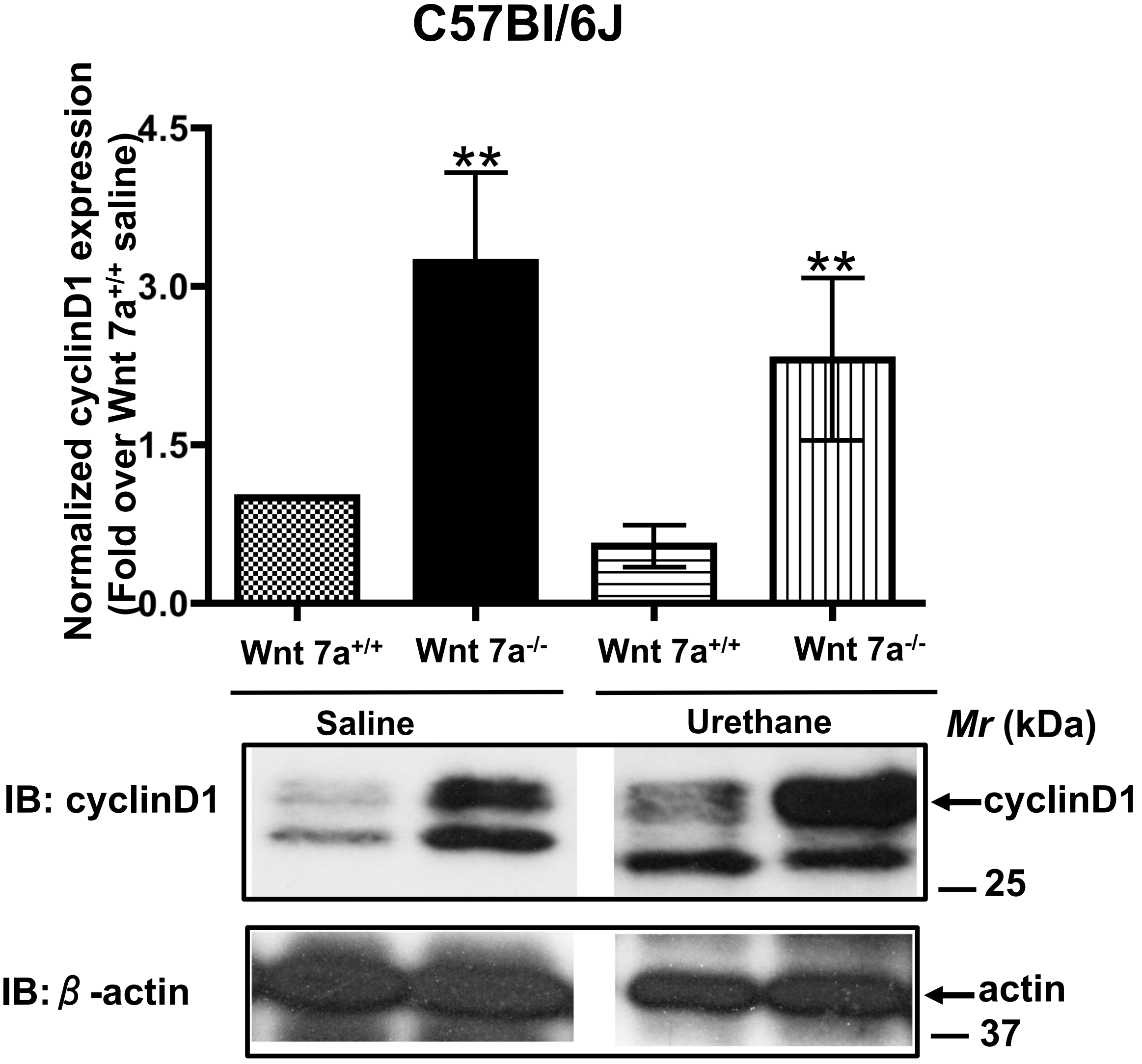

C57Bl/6J
Normalized cyclinD1 expression (Fold over Wnt 7a+/+ saline)
4.5
3.0
1.5
0.0
**
**
Wnt 7a+/+
Wnt 7a-/-
Wnt 7a+/+
Wnt 7a-/-
Saline
Urethane
Mr (kDa)
IB: cyclinD1
cyclinD1
25
IB: β -actin
actin
37

S5

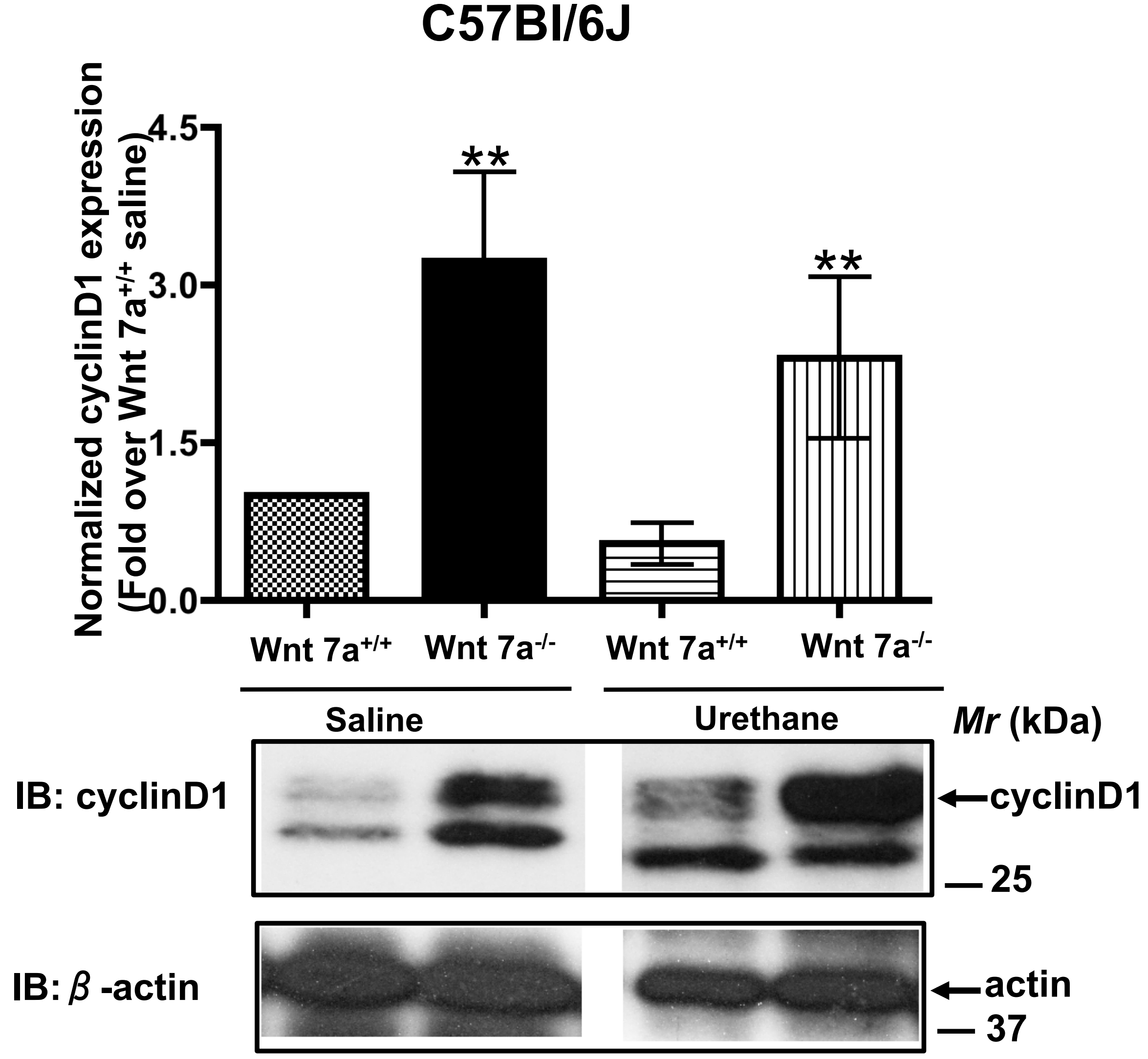
C57Bl/6J
Normalized cyclinD1 expression
(Fold over Wnt 7a+/+ saline)
4.5
3.0
1.5
0.0
**
**
Wnt 7a+/+
Wnt 7a-/-
Wnt 7a+/+
Wnt 7a-/-
Saline
Urethane
Mr (kDa)
IB: cyclinD1
cyclinD1
25
IB: β -actin
actin
37

S6

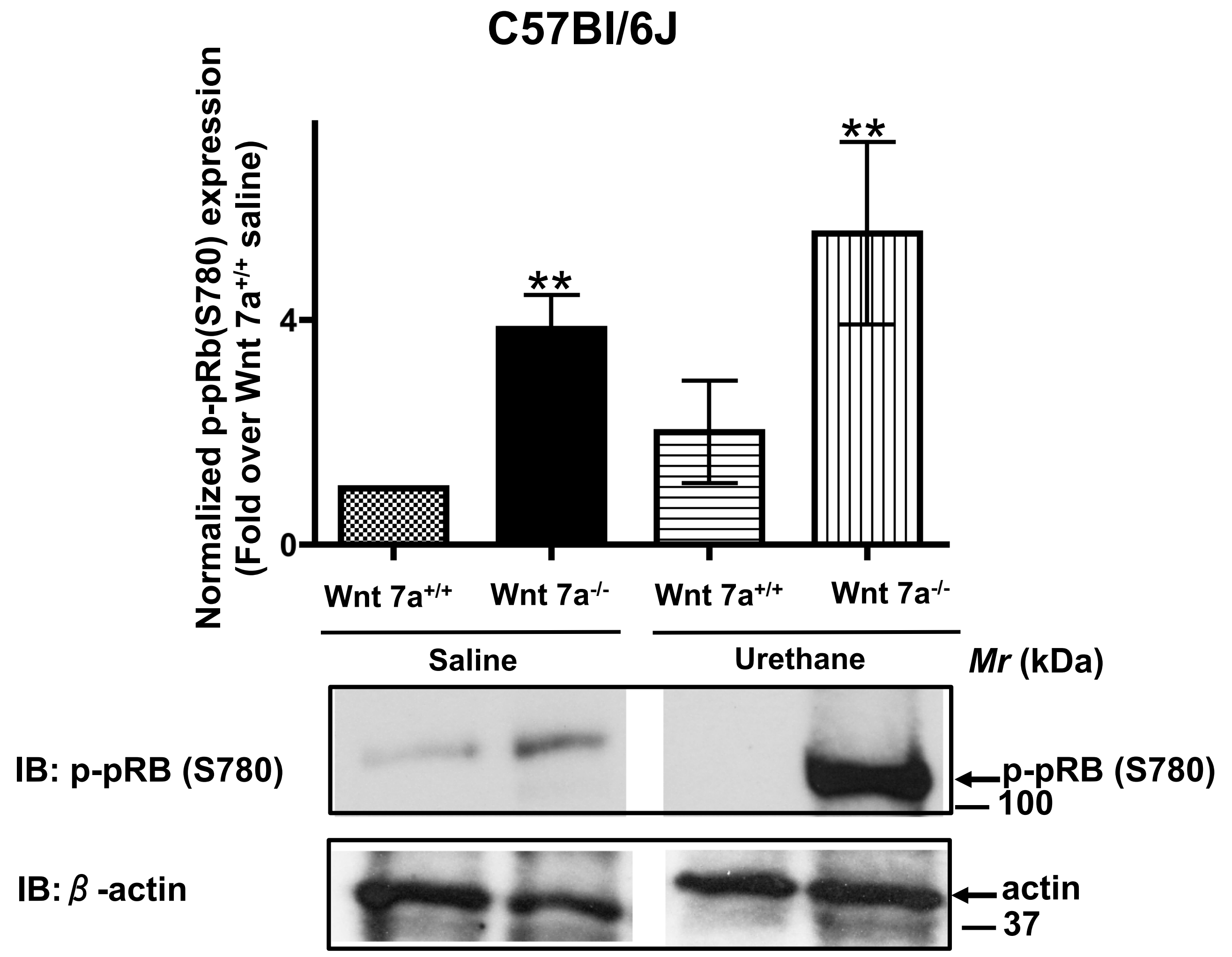
C57Bl/6J
Normalized p-pRb(S780) expression (Fold over Wnt 7a+/+ saline)
4
0
**
**
Wnt 7a+/+
Wnt 7a-/-
Wnt 7a+/+
Wnt 7a-/-
Saline
Urethane
Mr (kDa)
IB: p-pRB (S780)
p-pRB (S780)
100
IB: β -actin
actin
37

S7

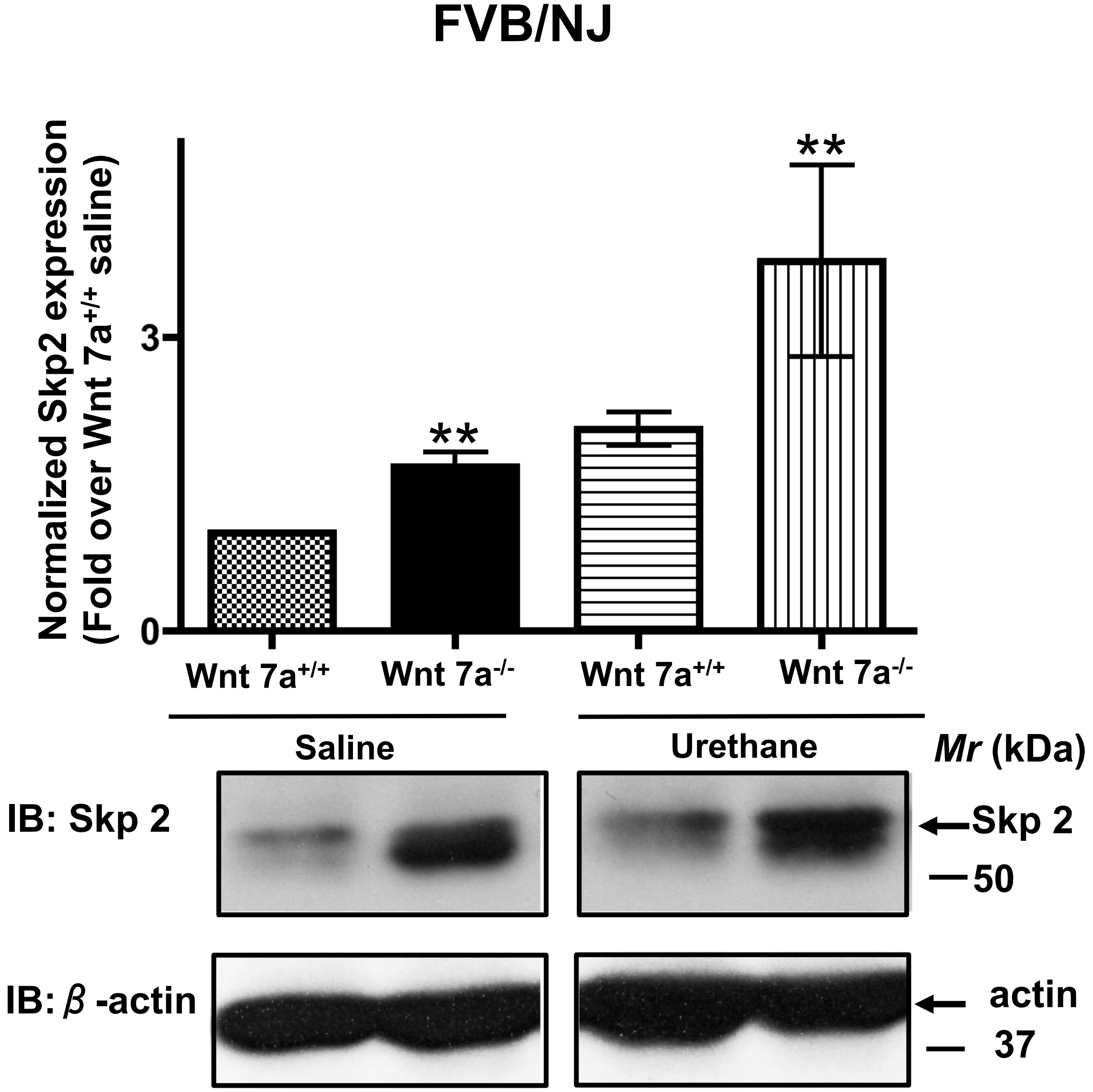
FVB/NJ
Normalized Skp2 expression (Fold over Wnt 7a+/+ saline)
3
0
**
**
Wnt 7a+/+
Wnt 7a-/-
Wnt 7a+/+
Wnt 7a-/-
Saline
Urethane
Mr (kDa)
IB: Skp 2
Skp 2
50
IB: β -actin
actin
37

S8

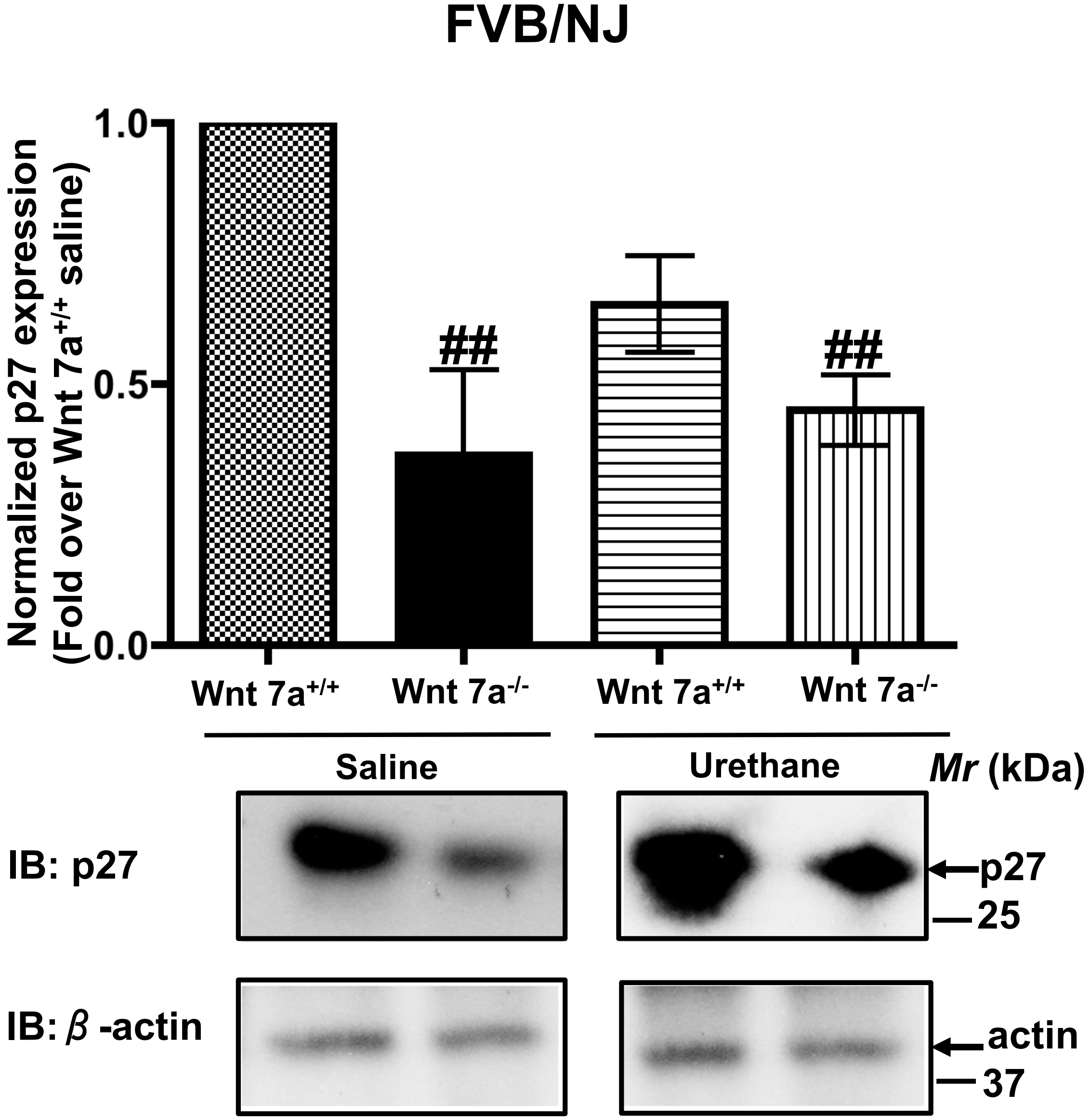
FVB/NJ
Normalized p27 expression
(Fold over Wnt 7a+/+ saline)
1.0
0.5
0.0
##
##
Wnt 7a+/+
Wnt 7a-/-
Wnt 7a+/+
Wnt 7a-/-
Saline
Urethane
Mr (kDa)
IB: p27
p27
25
IB: β -actin
actin
37

S9

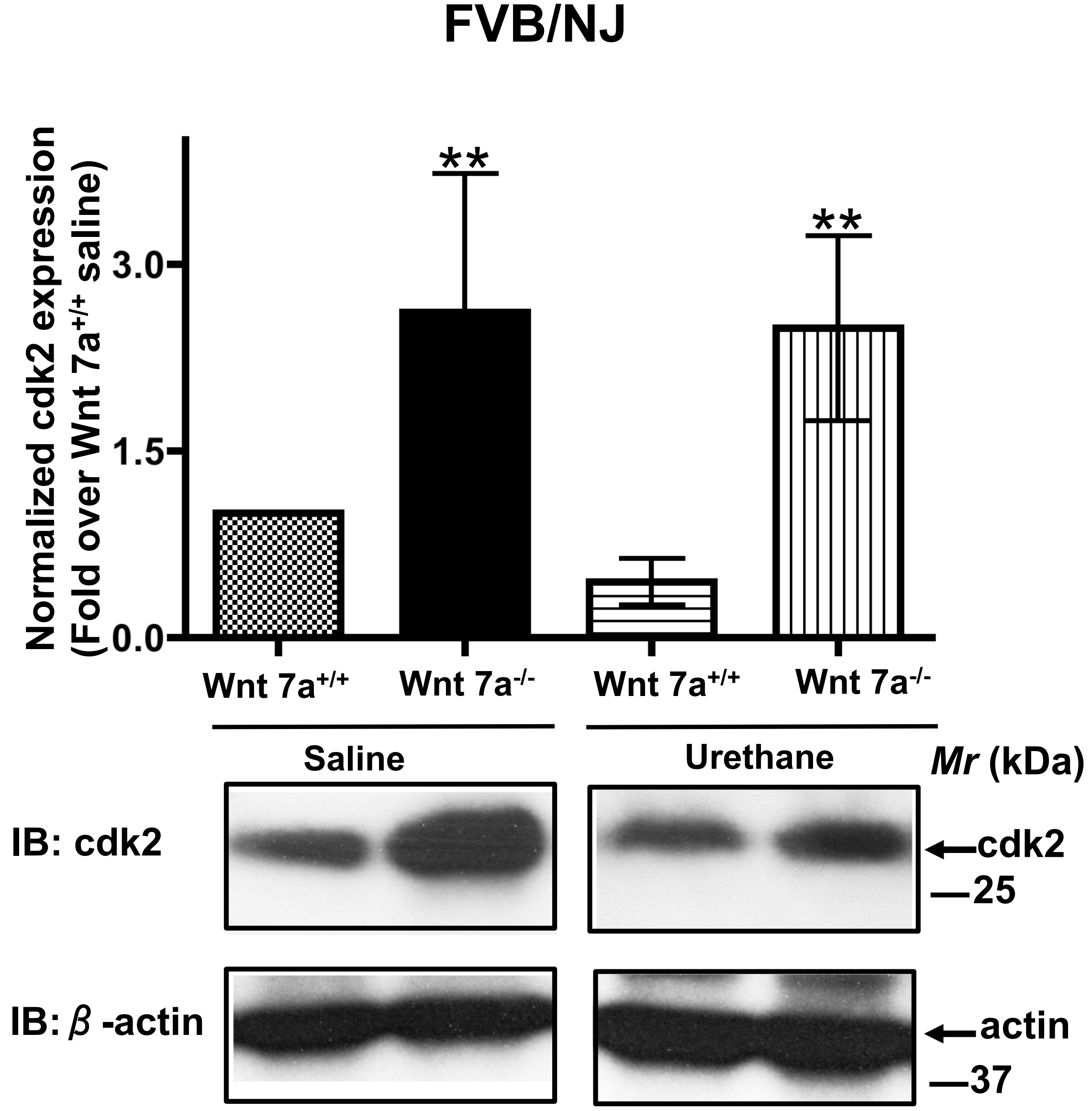
FVB/NJ
Normalized cdk2 expression
(Fold over Wnt 7a+/+ saline)
3.0
1.5
0.0
**
**
Wnt 7a+/+
Wnt 7a-/-
Wnt 7a+/+
Wnt 7a-/-
Saline
Urethane
Mr (kDa)
IB: cdk2
cdk2
25
IB: β -actin
actin
37

S10

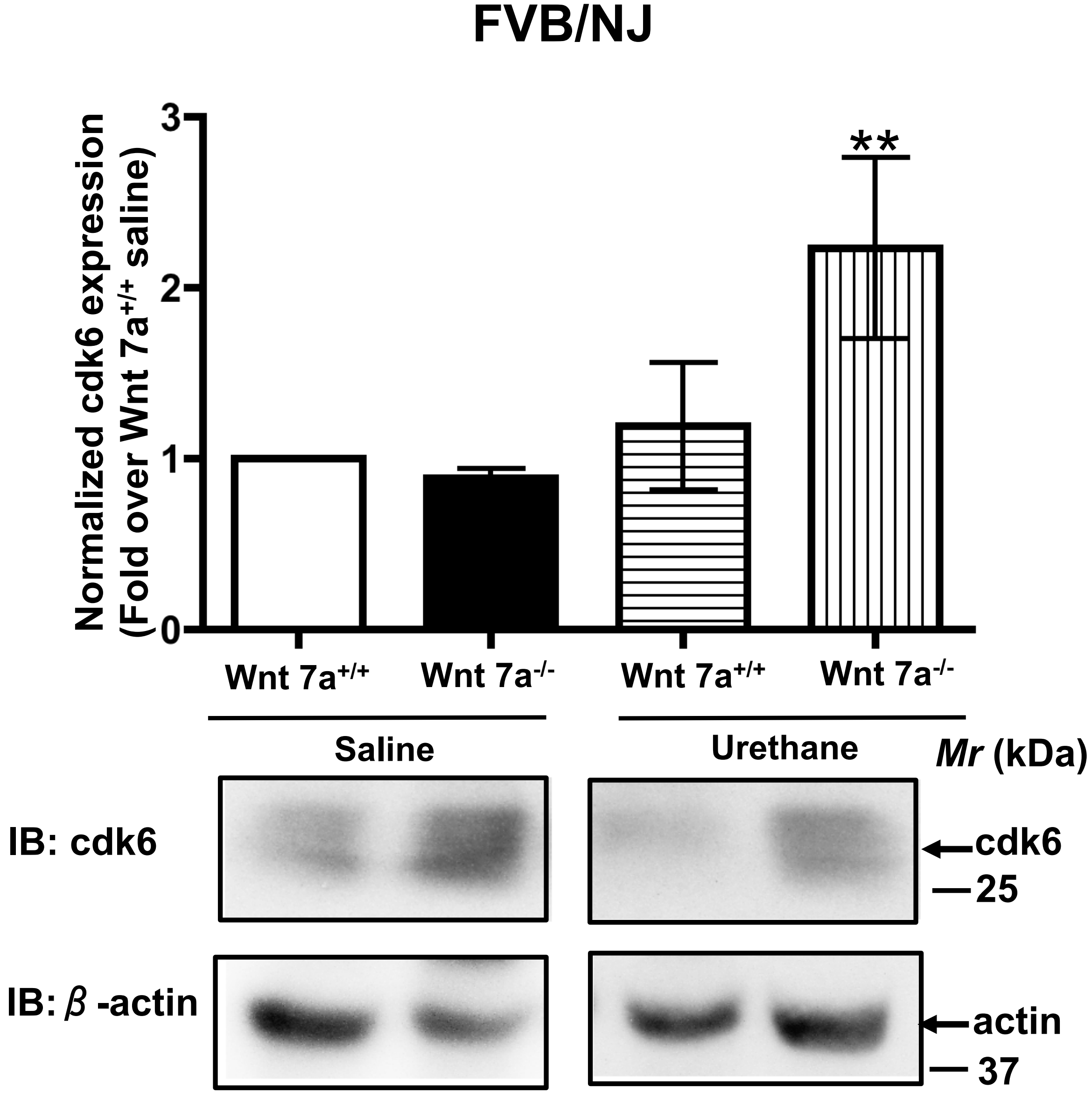

FVB/NJ
Normalized cdk6 expression
(Fold over Wnt 7a+/+ saline)
0
1
2
3
**
Wnt 7a+/+
Wnt 7a-/-
Wnt 7a+/+
Wnt 7a-/-
Saline
Urethane
Mr (kDa)
IB: cdk6
cdk6
25
IB: β -actin
actin
37

S11

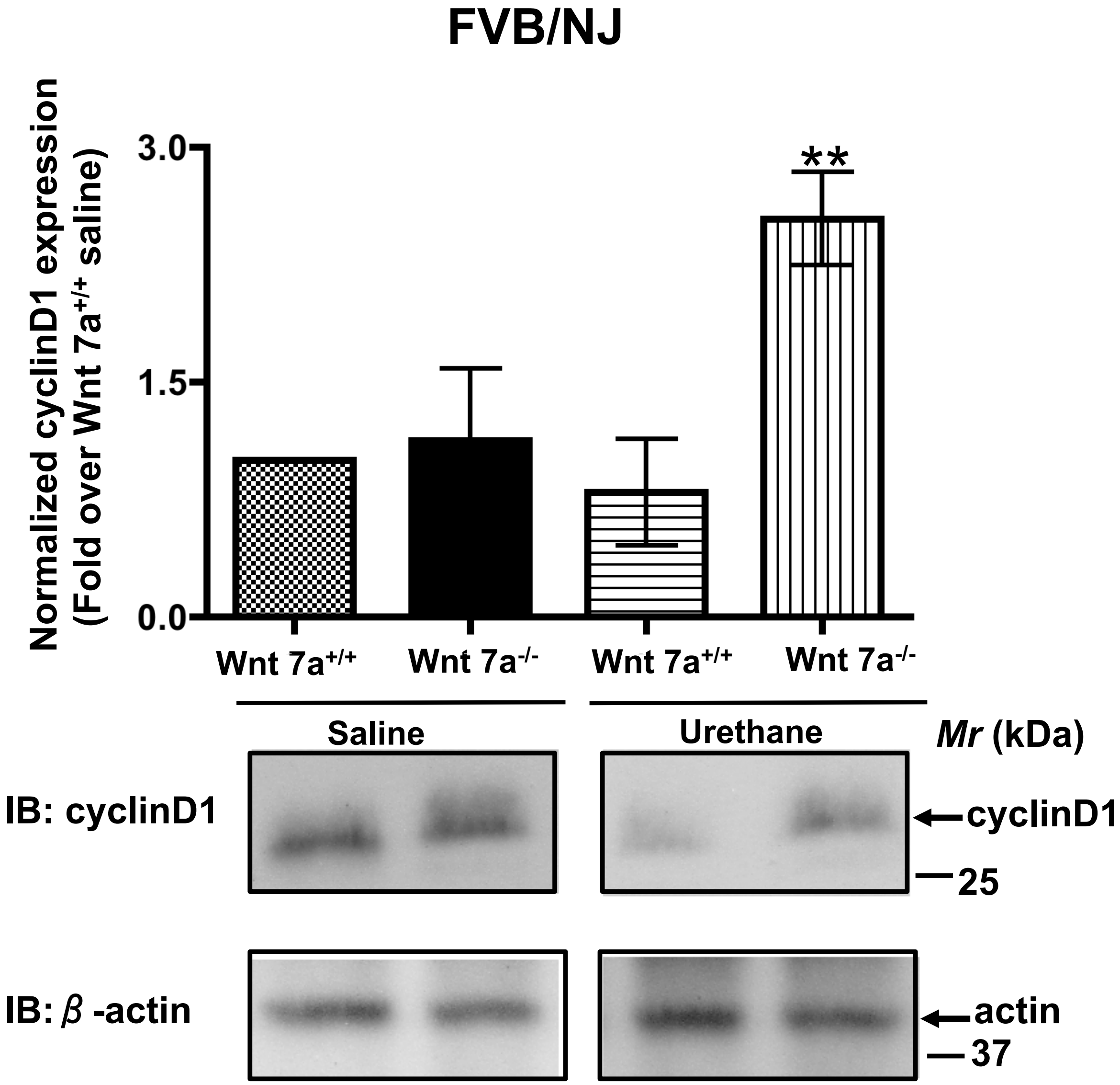

FVB/NJ
Normalized cyclinD1 expression
(Fold over Wnt 7a+/+ saline)
3.0
1.5
0.0
**
Wnt 7a+/+
Wnt 7a-/-
Wnt 7a+/+
Wnt 7a-/-
Saline
Urethane
Mr (kDa)
IB: cyclinD1
cyclinD1
25
IB: β -actin
actin
37

S12

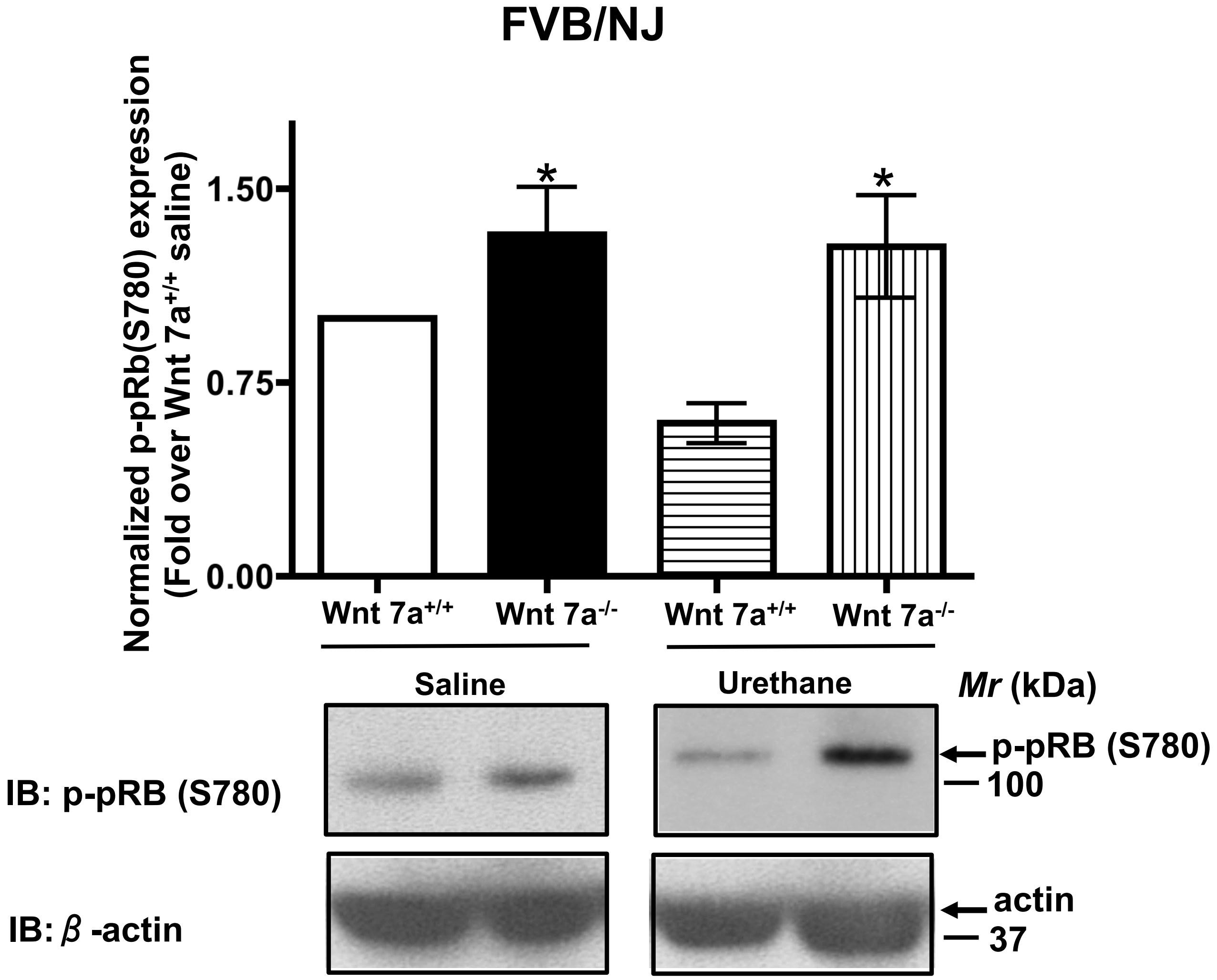
FVB/NJ
Normalized p-pRb(S780) expression (Fold over Wnt 7a+/+ saline)
1.50
0.75
0.00
*
*
Wnt 7a+/+
Wnt 7a-/-
Wnt 7a+/+
Wnt 7a-/-
Saline
Urethane
Mr (kDa)
IB: p-pRB (S780)
p-pRB (S780)
100
IB: β -actin
actin
37

S13

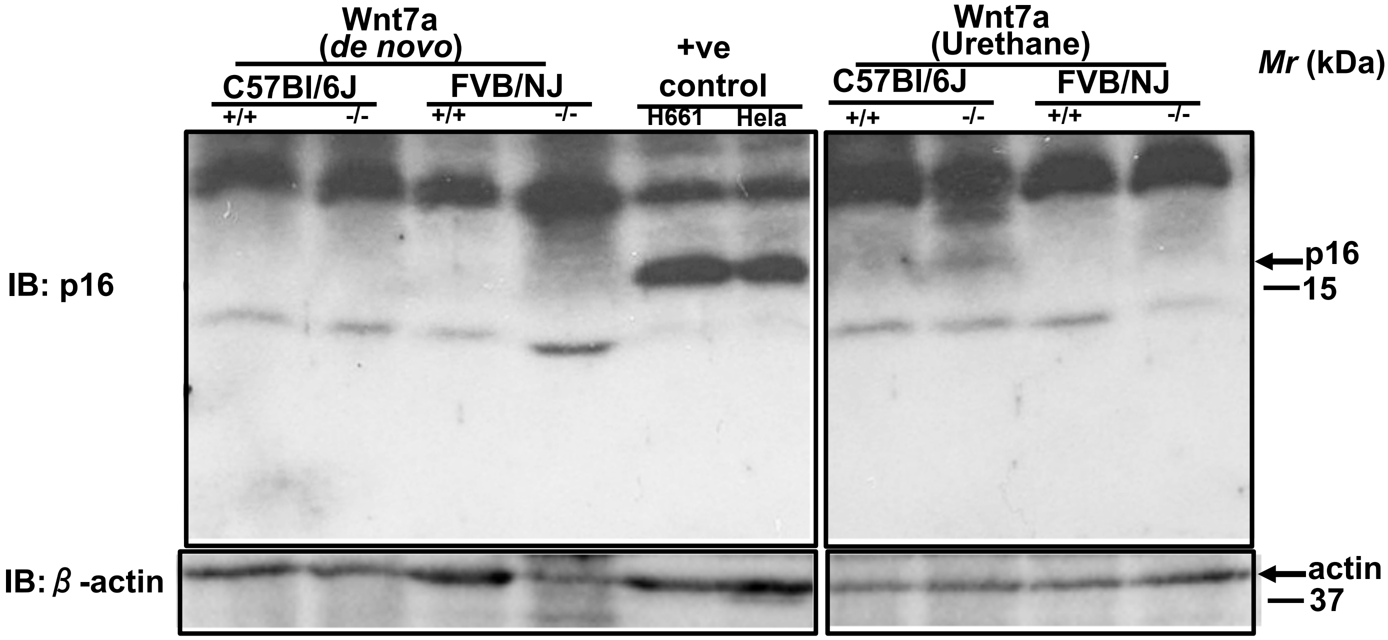

Wnt7a
(de novo)
C57Bl/6J
FVB/NJ
+/+
-/-
+/+
-/-
+ve
control
H661
Hela
Wnt7a
(Urethane)
C57Bl/6J
FVB/NJ
+/+
-/-
+/+
-/-
Mr (kDa)
IB: p16
p16
15
IB: β -actin
actin
37

S14

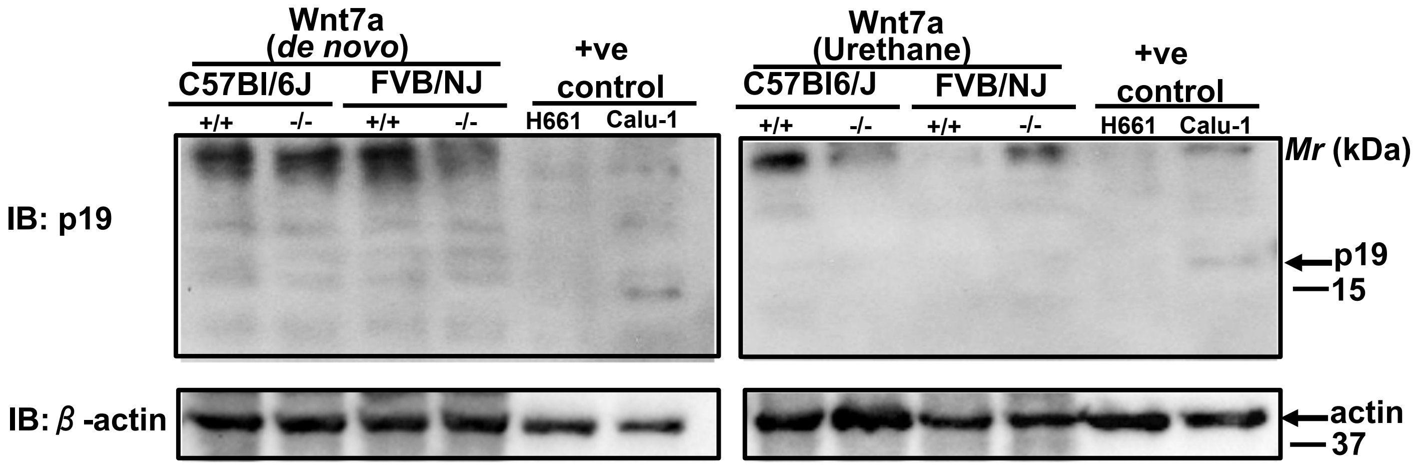
Wnt7a
(*de novo*)
C57Bl/6J
FVB/NJ
+/+
-/-
+/+
-/-
+ve
control
H661
Calu-1
Wnt7a
(Urethane)
C57Bl6/J
FVB/NJ
+/+
-/-
+/+
-/-
+ve
control
H661
Calu-1
Mr (kDa)
IB: p19
p19
15
IB: β -actin
actin
37
