# Supplementary material for: Wnt7a is a novel inducer of β-catenin-independent tumor-suppressive cellular senescence in lung cancer
Source: Oncogene. 2015 Mar 2;34(42):5317–28. doi: 10.1038/onc.2015.2 (PMC4558401; doi:10.1038/onc.2015.2)
Supplement: Supplementary Information [file onc20152x2.pdf]

Supplementary Table 1: List of Wnt7a-dependent genes regulating cellular senescence

S.No	Gene	Fold change	Full name	Reference	Function
1	Sox9	3.42022	HMG-box DNA binding protein	(1, 2)	Anti-senescence
2	Etv4	2.70759	Ets variant 4	(3)	
3	Trp63	2.56189	p63	(4)	
4	Tbx15	5.13303	T-box 15	(5-7)	
5	Asb11	2.13608	Ankyrin repeat and SOCS box containing 11		
6	Asb16	2.97284	Ankyrin repeat and SOCS box containing 16		
7	Asb5	5.35632	Ankyrin repeat and SOCS box containing 5		
8	Mt2	2.2828	Melatonin receptor B1	(8)	
9	Car12	3.36266	Car12 carbonic anyhydrase 12	(9)	
10	Krt13	8.60561	Keratin 13	(10)	
11	Zmat4	2.22248	Zinc finger, matrin-type 4	(11)	
12	Pygm	2.20326	Phosphorylase, glycogen, muscle	(12)	
13	Mt2	6.39376	Melatonin receptor B1	(8)	
14	Icam2	-1.44095	Intercellular adhesion molecule-1	(13, 14)	Pro-senescence
15	Ucp1	-7.46458	Uncoupling protein 1	(15)	
16	Saa3	-2.80801	Saa3 serum amyloid A 3	(16, 17)	
17	Cyyr1	-1.37799	Cysteine/tyrosine-rich 1	(18)	
18	Gap43	-2.33236	Gap-43 protein	(19)	
19	Mmp12	-1.9959	Matrix metallopeptidase 12 (macrophage elastase)	(20, 21)	
20	Rac2	-2.07098	Ras-related C3 botulinum toxin substrate 2	(22)	
21	Ddah1	-1.39073	Dimethylarginine dimethylaminohydrolase 1	(23)	
22	Cxcr5	-1.7861	CXCR5 chemokine (C-X-C motif) receptor 5		
23	Gbp2	-1.12508	Guanylate binding protein 2, interferon-inducible	(24, 25)	
24	Gbp10	-2.49643	guanylate-binding protein 10	(24)	
25	Foxf2	-1.37866	Forkhead box F2	(26, 27)	
26	Faim3	-2.0519	Fas apoptotic inhibitory molecule 3	(28)	

27	Ceacam 10	-2.44916	Carcinoembryonic antigen-related cell adhesion molecule 1	(29)	
28	Abcb1a	-1.36486	ATP-binding cassette, sub-family B (MDR/TAP), member 1A	(30)	
29	Il2rg	-1.17919	Interleukin 2 receptor, gamma	(31)	
30	Cdh5	-1.1364	Cadherin 5, Type 2 (Vascular Endothelium	(32)	

References:

1. Wang G, Lunardi A, Zhang J, Chen Z, Ala U, Webster KA, et al. Zbtb7a suppresses prostate cancer through repression of a Sox9-dependent pathway for cellular senescence bypass and tumor invasion. Nat Genet. 2013;45(7):739-46. Epub 2013/06/04.
2. Matheu A, Collado M, Wise C, Manterola L, Cekaite L, Tye AJ, et al. Oncogenicity of the developmental transcription factor Sox9. Cancer Res. 2012;72(5):1301-15. Epub 2012/01/17.
3. Huot TJ, Rowe J, Harland M, Drayton S, Brookes S, Gooptu C, et al. Biallelic mutations in p16(INK4a) confer resistance to Ras- and Ets-induced senescence in human diploid fibroblasts. Mol Cell Biol. 2002;22(23):8135-43. Epub 2002/11/06.
4. Keyes WM, Wu Y, Vogel H, Guo X, Lowe SW, Mills AA. p63 deficiency activates a program of cellular senescence and leads to accelerated aging. Genes Dev. 2005;19(17):1986-99. Epub 2005/08/19.
5. Abrahams A, Parker MI, Prince S. The T-box transcription factor Tbx2: its role in development and possible implication in cancer. IUBMB life. 2010;62(2):92-102. Epub 2009/12/05.
6. Carlson H, Ota S, Campbell CE, Hurlin PJ. A dominant repression domain in Tbx3 mediates transcriptional repression and cell immortalization: relevance to mutations in Tbx3 that cause ulnar-mammary syndrome. Hum Mol Genet. 2001;10(21):2403-13. Epub 2001/11/02.
7. Jacobs JJ, Keblusek P, Robanus-Maandag E, Kristel P, Lingbeek M, Nederlof PM, et al. Senescence bypass screen identifies TBX2, which represses Cdkn2a (p19(ARF)) and is amplified in a subset of human breast cancers. Nat Genet. 2000;26(3):291-9. Epub 2000/11/04.
8. Song N, Kim AJ, Kim HJ, Jee HJ, Kim M, Yoo YH, et al. Melatonin suppresses doxorubicin-induced premature senescence of A549 lung cancer cells by ameliorating mitochondrial dysfunction. Journal of pineal research. 2012;53(4):335-43. Epub 2012/04/28.
9. Wohlrab H, Bronson RT, Lu RC, Nemeth V. Towards a biomarker of mammalian senescence: carbonic anhydrase III. Biochem Biophys Res Commun. 1988;154(3):1130-6. Epub 1988/08/15.
10. Dickson MA, Hahn WC, Ino Y, Ronfard V, Wu JY, Weinberg RA, et al. Human keratinocytes that express hTERT and also bypass a p16(INK4a)-enforced mechanism that limits life span become immortal yet retain normal growth and differentiation characteristics. Mol Cell Biol. 2000;20(4):1436-47. Epub 2000/01/29.
11. Kim BC, Lee HC, Lee JJ, Choi CM, Kim DK, Lee JC, et al. Wig1 prevents cellular senescence by regulating p21 mRNA decay through control of RISC recruitment. EMBO J. 2012;31(22):4289-303. Epub 2012/10/23.

12. Ros S, Schulze A. Linking glycogen and senescence in cancer cells. Cell metabolism. 2012;16(6):687-8. Epub 2012/12/12.
13. Gorgoulis VG, Pratsinis H, Zacharatos P, Demoliou C, Sigala F, Asimacopoulos PJ, et al. p53-dependent ICAM-1 overexpression in senescent human cells identified in atherosclerotic lesions. Lab Invest. 2005;85(4):502-11. Epub 2005/02/16.
14. Luo H, Yang A, Schulte BA, Wargovich MJ, Wang GY. Resveratrol induces premature senescence in lung cancer cells via ROS-mediated DNA damage. PLoS One. 2013;8(3):e60065. Epub 2013/03/28.
15. Shimasaki Y, Pan N, Messina LM, Li C, Chen K, Liu L, et al. Uncoupling Protein 2 Impacts Endothelial Phenotype via p53-Mediated Control of Mitochondrial Dynamics. Circ Res. 2013;113(7):891-901. Epub 2013/07/04.
16. Chang BD, Watanabe K, Broude EV, Fang J, Poole JC, Kalinichenko TV, et al. Effects of p21Waf1/Cip1/Sdi1 on cellular gene expression: implications for carcinogenesis, senescence, and age-related diseases. Proc Natl Acad Sci U S A. 2000;97(8):4291-6. Epub 2000/04/13.
17. Yamada M, Tsukagoshi H, Otomo E, Hayakawa M. Systemic amyloid deposition in old age and dementia of Alzheimer type: the relationship of brain amyloid to other amyloid. Acta neuropathologica. 1988;77(2):136-41. Epub 1988/01/01.
18. Chan JM, Ho SH, Tai IT. Secreted protein acidic and rich in cysteine-induced cellular senescence in colorectal cancers in response to irinotecan is mediated by P53. Carcinogenesis. 2010;31(5):812-9. Epub 2010/02/19.
19. Zhao W, Lin ZX, Zhang ZQ. Cisplatin-induced premature senescence with concomitant reduction of gap junctions in human fibroblasts. Cell Res. 2004;14(1):60-6. Epub 2004/03/26.
20. Benanti JA, Williams DK, Robinson KL, Ozer HL, Galloway DA. Induction of extracellular matrix-remodeling genes by the senescence-associated protein APA-1. Mol Cell Biol. 2002;22(21):7385-97. Epub 2002/10/09.
21. Haynes KA, Silver PA. Synthetic reversal of epigenetic silencing. J Biol Chem. 2011;286(31):27176-82. Epub 2011/06/15.
22. Debidda M, Williams DA, Zheng Y. Rac1 GTPase regulates cell genomic stability and senescence. J Biol Chem. 2006;281(50):38519-28. Epub 2006/10/13.
23. Bode-Boger SM, Scalera F, Martens-Lobenhoffer J. Asymmetric dimethylarginine (ADMA) accelerates cell senescence. Vasc Med. 2005;10 Suppl 1:S65-71. Epub 2006/02/01.
24. Pammer J, Reinisch C, Birner P, Pogoda K, Sturzl M, Tschachler E. Interferon-alpha prevents apoptosis of endothelial cells after short-term exposure but induces replicative senescence after continuous stimulation. Lab Invest. 2006;86(10):997-1007. Epub 2006/08/09.
25. Vestal DJ, Jeyaratnam JA. The guanylate-binding proteins: emerging insights into the biochemical properties and functions of this family of large interferon-induced guanosine triphosphatase. Journal of interferon & cytokine research : the official journal of the International Society for Interferon and Cytokine Research. 2011;31(1):89-97. Epub 2010/12/15.
26. Wang IC, Chen YJ, Hughes D, Petrovic V, Major ML, Park HJ, et al. Forkhead box M1 regulates the transcriptional network of genes essential for mitotic progression and genes encoding the SCF (Skp2-Cks1) ubiquitin ligase. Mol Cell Biol. 2005;25(24):10875-94. Epub 2005/11/30.
27. de Keizer PL, Packer LM, Szypowska AA, Riedl-Polderman PE, van den Broek NJ, de Bruin A, et al. Activation of forkhead box O transcription factors by oncogenic BRAF promotes p21cip1-dependent senescence. Cancer Res. 2010;70(21):8526-36. Epub 2010/10/21.

28. Rebbaa A, Zheng X, Chou PM, Mirkin BL. Caspase inhibition switches doxorubicin-induced apoptosis to senescence. Oncogene. 2003;22(18):2805-11. Epub 2003/05/14.
29. Sappino AP, Buser R, Seguin Q, Fernet M, Lesne L, Gumy-Pause F, et al. The CEACAM1 tumor suppressor is an ATM and p53-regulated gene required for the induction of cellular senescence by DNA damage. Oncogenesis. 2012;1:e7. Epub 2012/01/01.
30. Wu B, Ueno M, Kusaka T, Onodera M, Huang CL, Hosomi N, et al. Abcb1a and Abcb1b expression in senescence-accelerated mouse (SAM). Neurosci Lett. 2009;456(1):34-8. Epub 2009/05/12.
31. Braumuller H, Wieder T, Brenner E, Assmann S, Hahn M, Alkhaled M, et al. T-helper-1-cell cytokines drive cancer into senescence. Nature. 2013;494(7437):361-5. Epub 2013/02/05.
32. Krouwer VJ, Hekking LH, Langelaar-Makkinje M, Regan-Klapisz E, Post JA. Endothelial cell senescence is associated with disrupted cell-cell junctions and increased monolayer permeability. Vascular cell. 2012;4(1):12. Epub 2012/08/30.

Supplementary Table 2: List of Wnt7a-dependent genes regulating EMT

S.No	Gene	Fold change	Full name	Reference
1	Sox9	3.42022	HMG-box DNA binding protein	[1]
2	Actn3	4.70638	Actinin, alpha 3	[2]
3	Mif2	4.33464	Antigen p97 (melanoma)	[3]
4	Fetub	1.63302	Fetun B	[4]
5	Pkp1	6.05813	Plakophilin 1	[5, 6]
6	Xirp2	3.86026	Xin actin-binding repeat containing 2	[7]
7	Lypd3	3.80475	LY6/PLAUR domain containing 3	[8]
8	Ntm	2.06434	Neurotrimin	[9]
9	Rap1gap	1.24152	RAP1 GTPase activating protein	[10]
10	Abra	2.55266	Actin-binding Rho activating protein	[11]
11	Gja3	3.37727	Gap junction protein 3	[12]
12	Plaa2g4e	3.26589	Phospholipase A2	[13]
13	Klk13	2.00867	Kallikerin-related peptidase 13	[14]
14	Adm2	2.88554	Adrenomodullin 2	[15]
15	Etv4	2.70759	Ets variant 4	[16],[17]
16	Pthlh	1.92246	Parathyroid harmone-like	[16]
17	Nos1	3.97291	Nitric oxide synthase 1	[18]
18	Spock2	-1.32681	Sparc/osteonectin	[19]
19	Tspan18	-1.22415	Tetraspanin 18	[20]
20	FoxF2	-1.37866	Forkhead box F2	[21]
21	Cdh5	-1.1364	Cadherin 5, Type 2	[22]
22	Cldn5	-1.13611	Claudin 5	[23],[24]
23	Spp1	-1.12632	Secreted phosphoprotein 1	[25]

References:

1. Acloque, H., et al., *Epithelial-mesenchymal transitions: the importance of changing cell state in development and disease.* J Clin Invest, 2009. **119**(6): p. 1438-49.
2. Ngan, E., et al., *A complex containing LPP and alpha-actinin mediates TGFbeta-induced migration and invasion of ErbB2-expressing breast cancer cells.* J Cell Sci, 2013. **126**(Pt 9): p. 1981-91.
3. Bonomi, S., et al., *Oncogenic alternative splicing switches: role in cancer progression and prospects for therapy.* Int J Cell Biol, 2013. **2013**: p. 962038.
4. Schliekelman, M.J., et al., *Targets of the tumor suppressor miR-200 in regulation of the epithelial-mesenchymal transition in cancer.* Cancer Res, 2011. **71**(24): p. 7670-82.

5. Chidgey, M. and C. Dawson, *Desmosomes: a role in cancer?* Br J Cancer, 2007. **96**(12): p. 1783-7.
6. Neuber, S., et al., *The desmosomal plaque proteins of the plakophilin family.* Dermatol Res Pract, 2010. **2010**: p. 101452.
7. Wang, Q., et al., *Xin proteins and intercalated disc maturation, signaling and diseases.* Front Biosci (Landmark Ed), 2012. **17**: p. 2566-93.
8. Oshiro, R., et al., *C4.4A is associated with tumor budding and epithelial-mesenchymal transition of colorectal cancer.* Cancer Sci, 2012. **103**(6): p. 1155-64.
9. Hesling, C., et al., *Tif1gamma is essential for the terminal differentiation of mammary alveolar epithelial cells and for lactation through SMAD4 inhibition.* Development, 2013. **140**(1): p. 167-75.
10. Mitra, R.S., et al., *Rap1GAP promotes invasion via induction of matrix metalloproteinase 9 secretion, which is associated with poor survival in low N-stage squamous cell carcinoma.* Cancer Res, 2008. **68**(10): p. 3959-69.
11. Clay, M.R. and M.C. Halloran, *Rho activation is apically restricted by Arhgap1 in neural crest cells and drives epithelial-to-mesenchymal transition.* Development, 2013. **140**(15): p. 3198-209.
12. Rhee, D.Y., et al., *Connexin 43 regulates epicardial cell polarity and migration in coronary vascular development.* Development, 2009. **136**(18): p. 3185-93.
13. Abalsamo, L., et al., *Inhibition of phosphatidylcholine-specific phospholipase C results in loss of mesenchymal traits in metastatic breast cancer cells.* Breast Cancer Res, 2012. **14**(2): p. R50.
14. Emami, N. and E.P. Diamandis, *New insights into the functional mechanisms and clinical applications of the kallikrein-related peptidase family.* Mol Oncol, 2007. **1**(3): p. 269-87.
15. Norregaard, R., et al., *Increased renal adrenomedullin expression in rats with ureteral obstruction.* Am J Physiol Regul Integr Comp Physiol, 2009. **296**(1): p. R185-92.
16. Kobberup, S., et al., *ETS-family genes in pancreatic development.* Dev Dyn, 2007. **236**(11): p. 3100-10.
17. Pellecchia, A., et al., *Overexpression of ETV4 is oncogenic in prostate cells through promotion of both cell proliferation and epithelial to mesenchymal transition.* Oncogenesis, 2012. **1**: p. e20.
18. Liu, F., A.M. Gomez Garcia, and F.L. Meyskens, Jr., *NADPH oxidase 1 overexpression enhances invasion via matrix metalloproteinase-2 and epithelial-mesenchymal transition in melanoma cells.* J Invest Dermatol, 2012. **132**(8): p. 2033-41.
19. Verma, M., P. Patel, and M. Verma, *Biomarkers in prostate cancer epidemiology.* Cancers (Basel), 2011. **3**(4): p. 3773-98.
20. Fairchild, C.L. and L.S. Gammill, *Tetraspanin18 is a FoxD3-responsive antagonist of cranial neural crest epithelial-to-mesenchymal transition that maintains cadherin-6B protein.* J Cell Sci, 2013. **126**(Pt 6): p. 1464-76.
21. Aitola, M., et al., *Forkhead transcription factor FoxF2 is expressed in mesodermal tissues involved in epithelio-mesenchymal interactions.* Dev Dyn, 2000. **218**(1): p. 136-49.
22. van Roy, F., *Beyond E-cadherin: roles of other cadherin superfamily members in cancer.* Nat Rev Cancer, 2014. **14**(2): p. 121-34.
23. Overgaard, C.E., et al., *Claudins: control of barrier function and regulation in response to oxidant stress.* Antioxid Redox Signal, 2011. **15**(5): p. 1179-93.

24. Kokudo, T., et al., *Snail is required for TGFbeta-induced endothelial-mesenchymal transition of embryonic stem cell-derived endothelial cells.* J Cell Sci, 2008. **121**(Pt 20): p. 3317-24.
25. Li, N.Y., et al., *Osteopontin up-regulates critical epithelial-mesenchymal transition transcription factors to induce an aggressive breast cancer phenotype.* J Am Coll Surg, 2013. **217**(1): p. 17-26; discussion 26.
