# Supplementary material for: Wnt7a is a novel inducer of β-catenin-independent tumor-suppressive cellular senescence in lung cancer
Source: Oncogene. 2015 Mar 2;34(42):5317–28. doi: 10.1038/onc.2015.2 (PMC4558401; doi:10.1038/onc.2015.2)
Supplement: Supplementary Table [file onc20152x3.pdf]

test_id	gene_id	gene	locus	sample_1	sample_2	status	value_1	value_2
ENSMUSG0	ENSMUSG0	Bcl6b	chr11:7003	q1	q2	OK	20.0331	7.52875
ENSMUSG0	ENSMUSG0	Sox9	chr11:1126	q1	q2	OK	0.554291	5.9337
ENSMUSG0	ENSMUSG0	Icam2	chr11:1062	q1	q2	OK	164.129	60.4528
ENSMUSG0	ENSMUSG0	Cnn1	chr9:21903	q1	q2	OK	14.8249	43.405
ENSMUSG0	ENSMUSG0	Col18a1	chr10:7649	q1	q2	OK	10.2895	39.7096
ENSMUSG0	ENSMUSG0	Aif1l	chr2:31805	q1	q2	OK	2.58129	8.66929
ENSMUSG0	ENSMUSG0	Rpl3l	chr17:2486	q1	q2	OK	0.959172	7.85385
ENSMUSG0	ENSMUSG0	Cd79a	chr7:25682	q1	q2	OK	28.0567	7.01848
ENSMUSG0	ENSMUSG0	Crhr2	chr6:55040	q1	q2	OK	0.191388	1.5009
ENSMUSG0	ENSMUSG0	Nes	chr3:87775	q1	q2	OK	25.3352	10.4399
ENSMUSG0	ENSMUSG0	Myl10	chr5:13716	q1	q2	OK	0	0.6365
ENSMUSG0	ENSMUSG0	Hspb7	chr4:14097	q1	q2	OK	7.2241	22.0193
ENSMUSG0	ENSMUSG0	Actn3	chr19:4861	q1	q2	OK	0.677508	17.6879
ENSMUSG0	ENSMUSG0	Slc4a1	chr11:1022	q1	q2	OK	1.37282	6.36845
ENSMUSG0	ENSMUSG0	Casq1	chr1:17414	q1	q2	OK	1.29096	17.4387
ENSMUSG0	ENSMUSG0	Tcap	chr11:9824	q1	q2	OK	28.5512	185.927
ENSMUSG0	ENSMUSG0	Spib	chr7:51781	q1	q2	OK	13.3044	3.94207
ENSMUSG0	ENSMUSG0	Slc38a3	chr9:10755	q1	q2	OK	0.115701	1.48099
ENSMUSG0	ENSMUSG0	Adssl1	chr12:1138	q1	q2	OK	21.2256	68.7022
ENSMUSG0	ENSMUSG0	Pcolce2	chr9:95538	q1	q2	OK	27.8491	11.2216
ENSMUSG0	ENSMUSG0	Cd5l	chr3:87161	q1	q2	OK	3.22892	12.6831
ENSMUSG0	ENSMUSG0	Sertad4	chr1:19467	q1	q2	OK	2.73783	7.54037
ENSMUSG0	ENSMUSG0	Eef1a2	chr2:18088	q1	q2	OK	4.72108	17.1872
ENSMUSG0	ENSMUSG0	Etv4	chr11:1016	q1	q2	OK	1.33883	8.74565
ENSMUSG0	ENSMUSG0	Ros1	chr10:5176	q1	q2	OK	0.963957	5.08405
ENSMUSG0	ENSMUSG0	Mybpc1	chr10:8798	q1	q2	OK	0.563449	23.5792
ENSMUSG0	ENSMUSG0	Mypn	chr10:6257	q1	q2	OK	0.316188	2.26476
ENSMUSG0	ENSMUSG0	Jsrp1	chr10:8027	q1	q2	OK	0.265752	9.17086
ENSMUSG0	ENSMUSG0	Pgam2	chr11:5701	q1	q2	OK	16.8725	61.7874
ENSMUSG0	ENSMUSG0	Slc26a4	chr12:3220	q1	q2	OK	34.2029	12.0898
ENSMUSG0	ENSMUSG0	Pitx1	chr13:5592	q1	q2	OK	0.372736	3.17115
ENSMUSG0	ENSMUSG0	Ldb3	chr14:3533	q1	q2	OK	5.05434	26.5345
ENSMUSG0	ENSMUSG0	Gdf10	chr14:3473	q1	q2	OK	3.91384	10.384
ENSMUSG0	ENSMUSG0	Trp63	chr16:2568	q1	q2	OK	0.405952	2.39707
ENSMUSG0	ENSMUSG0	Srl	chr16:4480	q1	q2	OK	3.65784	12.3504

ENSMUSG0	ENSMUSG0	Mfi2	chr16:3187	q1	q2	OK	0.0595272	1.20108
ENSMUSG0	ENSMUSG0	Ahsg	chr16:2289	q1	q2	OK	0.29262	2.45289
ENSMUSG0	ENSMUSG0	Fetub	chr16:2291	q1	q2	OK	11.922	36.9774
ENSMUSG0	ENSMUSG0	Myom1	chr17:7136	q1	q2	OK	4.03458	17.9644
ENSMUSG0	ENSMUSG0	Fkbp5	chr17:2853	q1	q2	OK	6.60941	16.7554
ENSMUSG0	ENSMUSG0	Myot	chr18:4449	q1	q2	OK	0.400021	24.4675
ENSMUSG0	ENSMUSG0	Cidea	chr18:6750	q1	q2	OK	29.5887	8.00449
ENSMUSG0	ENSMUSG0	Ms4a1	chr19:1132	q1	q2	OK	25.7943	4.50415
ENSMUSG0	ENSMUSG0	Col17a1	chr19:4772	q1	q2	OK	0.550217	2.44148
ENSMUSG0	ENSMUSG0	Sema4g	chr19:4506	q1	q2	OK	1.42564	4.10735
ENSMUSG0	ENSMUSG0	Pkp1	chr1:13776	q1	q2	OK	0.040442	2.69471
ENSMUSG0	ENSMUSG0	Dusp27	chr1:16802	q1	q2	OK	0.142329	1.13086
ENSMUSG0	ENSMUSG0	Neb	chr2:51992	q1	q2	OK	0.519038	23.0151
ENSMUSG0	ENSMUSG0	Xirp2	chr2:67284	q1	q2	OK	1.38287	20.0834
ENSMUSG0	ENSMUSG0	Stk39	chr2:68048	q1	q2	OK	6.4879	16.3398
ENSMUSG0	ENSMUSG0	Prg2	chr2:84820	q1	q2	OK	3.34755	16.4359
ENSMUSG0	ENSMUSG0	Mylk2	chr2:15273	q1	q2	OK	0.226073	5.40336
ENSMUSG0	ENSMUSG0	Pck1	chr2:17297	q1	q2	OK	26.5912	5.50675
ENSMUSG0	ENSMUSG0	Wisp2	chr2:16364	q1	q2	OK	37.3463	12.5091
ENSMUSG0	ENSMUSG0	Tbx15	chr3:99057	q1	q2	OK	0.100697	3.53358
ENSMUSG0	ENSMUSG0	Sypl2	chr3:10801	q1	q2	OK	0.278644	2.39887
ENSMUSG0	ENSMUSG0	Gucy1b3	chr3:81835	q1	q2	OK	23.4765	9.81932
ENSMUSG0	ENSMUSG0	Egf	chr3:12938	q1	q2	OK	0.301392	1.7355
ENSMUSG0	ENSMUSG0	Clca3	chr3:14466	q1	q2	OK	27.3734	6.13016
ENSMUSG0	ENSMUSG0	Murc	chr4:48676	q1	q2	OK	0.653246	4.76546
ENSMUSG0	ENSMUSG0	Zfp618	chr4:62626	q1	q2	OK	0.43781	1.69665
ENSMUSG0	ENSMUSG0	2310002L0	chr4:73585	q1	q2	OK	0.487072	4.45405
ENSMUSG0	ENSMUSG0	Chrna9	chr5:66326	q1	q2	OK	0.207473	4.56057
ENSMUSG0	ENSMUSG0	Gbp9	chr5:10550	q1	q2	OK	16.1932	6.45006
ENSMUSG0	ENSMUSG0	Plac8	chr5:10098	q1	q2	OK	179.758	55.5886
ENSMUSG0	ENSMUSG0	Nos1	chr5:11829	q1	q2	OK	0.0438104	0.687929
ENSMUSG0	ENSMUSG0	Alb	chr5:90889	q1	q2	OK	0.671734	8.16176
ENSMUSG0	ENSMUSG0	Cxcl5	chr5:91188	q1	q2	OK	28.2561	6.67129
ENSMUSG0	ENSMUSG0	Pf4	chr5:91201	q1	q2	OK	65.8907	180.305
ENSMUSG0	ENSMUSG0	Ereg	chr5:91503	q1	q2	OK	1.27361	6.45195
ENSMUSG0	ENSMUSG0	Lmod2	chr6:24547	q1	q2	OK	2.50857	12.6272

ENSMUSG0	ENSMUSG0	Cyp3a13	chr5:13833	q1	q2	OK	0.740425	2.89417
ENSMUSG0	ENSMUSG0	Trpv6	chr6:41570	q1	q2	OK	3.44048	9.90869
ENSMUSG0	ENSMUSG0	Gkn2	chr6:87323	q1	q2	OK	4.34805	125.476
ENSMUSG0	ENSMUSG0	Ckm	chr7:19996	q1	q2	OK	14.8447	367.397
ENSMUSG0	ENSMUSG0	Sbk2	chr7:49086	q1	q2	OK	0.636739	10.1195
ENSMUSG0	ENSMUSG0	Csrp3	chr7:56085	q1	q2	OK	29.6988	181.925
ENSMUSG0	ENSMUSG0	Plin1	chr7:86866	q1	q2	OK	12.8051	2.52344
ENSMUSG0	ENSMUSG0	Ryr1	chr7:29788	q1	q2	OK	0.230827	2.6115
ENSMUSG0	ENSMUSG0	Mylpf	chr7:13435	q1	q2	OK	10.1076	675.69
ENSMUSG0	ENSMUSG0	Cox6a2	chr7:13529	q1	q2	OK	55.8996	175.034
ENSMUSG0	ENSMUSG0	Tnni2	chr7:14962	q1	q2	OK	12.0733	514.12
ENSMUSG0	ENSMUSG0	Itgb1bp2	chrX:98644	q1	q2	OK	3.05153	21.0794
ENSMUSG0	ENSMUSG0	Asb11	chrX:16087	q1	q2	OK	1.96299	8.62861
ENSMUSG0	ENSMUSG0	Myom2	chr8:15057	q1	q2	OK	2.54441	9.90609
ENSMUSG0	ENSMUSG0	Gpm6a	chr8:56040	q1	q2	OK	26.0381	11.1732
ENSMUSG0	ENSMUSG0	Asb5	chr8:55635	q1	q2	OK	0.352463	14.4386
ENSMUSG0	ENSMUSG0	Pdlim3	chr8:46970	q1	q2	OK	14.0403	38.6511
ENSMUSG0	ENSMUSG0	Ucp1	chr8:85814	q1	q2	OK	43.8264	0.248126
ENSMUSG0	ENSMUSG0	Mt2	chr8:96696	q1	q2	OK	94.8623	461.62
ENSMUSG0	ENSMUSG0	Mt1	chr8:96702	q1	q2	OK	251.871	690.006
ENSMUSG0	ENSMUSG0	Tmem38a	chr8:75096	q1	q2	OK	8.60019	31.4228
ENSMUSG0	ENSMUSG0	Pde4c	chr8:73247	q1	q2	OK	1.98948	6.98172
ENSMUSG0	ENSMUSG0	Car12	chr9:66561	q1	q2	OK	0.141079	1.4512
ENSMUSG0	ENSMUSG0	Fhl3	chr4:12437	q1	q2	OK	4.38777	14.1785
ENSMUSG0	ENSMUSG0	Pygm	chr19:6384	q1	q2	OK	10.719	49.3628
ENSMUSG0	ENSMUSG0	Gucy1a3	chr3:81896	q1	q2	OK	23.1083	8.28548
ENSMUSG0	ENSMUSG0	Kcnk1	chr8:12851	q1	q2	OK	11.4473	32.5717
ENSMUSG0	ENSMUSG0	Ypel4	chr2:84574	q1	q2	OK	0.904469	6.67653
ENSMUSG0	ENSMUSG0	Lrrn1	chr6:10747	q1	q2	OK	0.114907	1.26706
ENSMUSG0	ENSMUSG0	Tnfsf9	chr17:5723	q1	q2	OK	14.0009	49.7667
ENSMUSG0	ENSMUSG0	Thrsp	chr7:10456	q1	q2	OK	66.4127	22.0537
ENSMUSG0	ENSMUSG0	Myom3	chr4:13531	q1	q2	OK	0.818595	3.1487
ENSMUSG0	ENSMUSG0	Rtkn2	chr10:6744	q1	q2	OK	69.0156	29.0246
ENSMUSG0	ENSMUSG0	Itln1	chr1:17344	q1	q2	OK	65.7288	15.7034
ENSMUSG0	ENSMUSG0	Hrc	chr7:52590	q1	q2	OK	3.24524	10.112
ENSMUSG0	ENSMUSG0	Hfe2	chr3:96329	q1	q2	OK	1.59626	8.27076

ENSMUSG0	ENSMUSG0	Akap12	chr10:5987	q1	q2	OK	22.0767	9.84188
ENSMUSG0	ENSMUSG0	Slc35f1	chr10:5241	q1	q2	OK	0.139936	1.01675
ENSMUSG0	ENSMUSG0	Mybpc2	chr7:51757	q1	q2	OK	1.63256	13.4522
ENSMUSG0	ENSMUSG0	Synpo2l	chr14:2147	q1	q2	OK	1.00235	5.21976
ENSMUSG0	ENSMUSG0	Txlnb	chr10:1751	q1	q2	OK	1.14815	5.71118
ENSMUSG0	ENSMUSG0	Saa3	chr7:53967	q1	q2	OK	485.449	69.3181
ENSMUSG0	ENSMUSG0	Apobec2	chr17:4855	q1	q2	OK	4.66721	81.4664
ENSMUSG0	ENSMUSG0	Cyyr1	chr16:8542	q1	q2	OK	14.1575	5.44715
ENSMUSG0	ENSMUSG0	Elovl6	chr3:12923	q1	q2	OK	4.96415	1.50781
ENSMUSG0	ENSMUSG0	Rap1gap	chr4:13722	q1	q2	OK	15.6857	37.0885
ENSMUSG0	ENSMUSG0	Smpx	chrX:15413	q1	q2	OK	8.37023	60.7837
ENSMUSG0	ENSMUSG0	Prelp	chr1:13580	q1	q2	OK	59.5089	24.9478
ENSMUSG0	ENSMUSG0	Lce3b	chr3:92736	q1	q2	OK	0	1.36604
ENSMUSG0	ENSMUSG0	Sln	chr9:53698	q1	q2	OK	86.7505	422.07
ENSMUSG0	ENSMUSG0	Slc26a9	chr1:13364	q1	q2	OK	17.6352	50.8244
ENSMUSG0	ENSMUSG0	S100a14	chr3:90330	q1	q2	OK	33.6567	83.1806
ENSMUSG0	ENSMUSG0	Ppp1r3a	chr6:14663	q1	q2	OK	0.71274	3.09895
ENSMUSG0	ENSMUSG0	Bex2	chrX:13260	q1	q2	OK	10.0185	40.7769
ENSMUSG0	ENSMUSG0	Trim72	chr7:13514	q1	q2	OK	1.51903	7.52278
ENSMUSG0	ENSMUSG0	Abra	chr15:4169	q1	q2	OK	0.96861	5.68296
ENSMUSG0	ENSMUSG0	Upk3b	chr5:13651	q1	q2	OK	46.2646	15.4311
ENSMUSG0	ENSMUSG0	Lor	chr3:91884	q1	q2	OK	0.166374	2.22131
ENSMUSG0	ENSMUSG0	Fam25c	chr14:3516	q1	q2	OK	1.86201	26.5774
ENSMUSG0	ENSMUSG0	Krt13	chr11:9997	q1	q2	OK	0.32864	128.017
ENSMUSG0	ENSMUSG0	Lmod3	chr6:97188	q1	q2	OK	0.425068	4.50328
ENSMUSG0	ENSMUSG0	Klhl31	chr9:77484	q1	q2	OK	0.284538	2.62767
ENSMUSG0	ENSMUSG0	Mylk4	chr13:3279	q1	q2	OK	0.566007	2.42991
ENSMUSG0	ENSMUSG0	Krt14	chr11:1000	q1	q2	OK	0.393587	34.4686
ENSMUSG0	ENSMUSG0	Gap43	chr16:4224	q1	q2	OK	16.8454	3.3448
ENSMUSG0	ENSMUSG0	Cmya5	chr13:9381	q1	q2	OK	0.747492	5.64651
ENSMUSG0	ENSMUSG0	Fbxo40	chr16:3696	q1	q2	OK	0.44753	1.7172
ENSMUSG0	ENSMUSG0	Bex4	chrX:13267	q1	q2	OK	3.70224	13.6735
ENSMUSG0	ENSMUSG0	Gja3	chr14:5765	q1	q2	OK	0.197774	2.05509
ENSMUSG0	ENSMUSG0	Pla2g4e	chr2:11999	q1	q2	OK	0.0587698	0.565308
ENSMUSG0	ENSMUSG0	Gypa	chr8:83017	q1	q2	OK	1.54299	6.93189
ENSMUSG0	ENSMUSG0	Actn2	chr13:1236	q1	q2	OK	6.18086	16.7209

ENSMUSG0	ENSMUSG0	Prx	chr7:28284	q1	q2	OK	41.495	17.2717
ENSMUSG0	ENSMUSG0	Lgals7	chr7:29648	q1	q2	OK	1.45481	147.955
ENSMUSG0	ENSMUSG0	Klk1b26	chr7:51268	q1	q2	OK	0	0.586274
ENSMUSG0	ENSMUSG0	Klk13	chr7:50967	q1	q2	OK	5.16308	20.7768
ENSMUSG0	ENSMUSG0	Adm2	chr15:8915	q1	q2	OK	0.597712	4.417
ENSMUSG0	ENSMUSG0	Krt15	chr11:9999	q1	q2	OK	2.9719	47.5124
ENSMUSG0	ENSMUSG0	Adh7	chr3:13788	q1	q2	OK	3.15527	11.2978
ENSMUSG0	ENSMUSG0	Myh8	chr11:6709	q1	q2	OK	0.142767	100.893
ENSMUSG0	ENSMUSG0	Myh1	chr11:6698	q1	q2	OK	1.33529	85.6408
ENSMUSG0	ENSMUSG0	Ptprn2	chr12:1177	q1	q2	OK	1.21966	4.17237
ENSMUSG0	ENSMUSG0	Spock2	chr10:5956	q1	q2	OK	93.3737	37.2233
ENSMUSG0	ENSMUSG0	Defb4	chr8:19198	q1	q2	OK	0	1.65624
ENSMUSG0	ENSMUSG0	Krt4	chr15:1017	q1	q2	OK	0.26799	52.4961
ENSMUSG0	ENSMUSG0	Myl3	chr9:11066	q1	q2	OK	6.54842	163.649
ENSMUSG0	ENSMUSG0	Kng2	chr16:2298	q1	q2	OK	4.26812	20.638
ENSMUSG0	ENSMUSG0	Krt5	chr15:1015	q1	q2	OK	0.898275	61.2254
ENSMUSG0	ENSMUSG0	Myl1	chr1:66970	q1	q2	OK	9.23341	95.2077
ENSMUSG0	ENSMUSG0	Trim54	chr5:31419	q1	q2	OK	1.35469	8.12946
ENSMUSG0	ENSMUSG0	Kcnc3	chr7:51846	q1	q2	OK	12.0082	35.035
ENSMUSG0	ENSMUSG0	Sox7	chr14:6456	q1	q2	OK	32.102	13.1209
ENSMUSG0	ENSMUSG0	Calml3	chr13:3802	q1	q2	OK	10.4961	31.1726
ENSMUSG0	ENSMUSG0	Uty	chrY:43330	q1	q2	OK	0.171708	1.55901
ENSMUSG0	ENSMUSG0	Myoz1	chr14:2146	q1	q2	OK	0.600559	35.1816
ENSMUSG0	ENSMUSG0	Flnc	chr6:29383	q1	q2	OK	0.985619	6.69845
ENSMUSG0	ENSMUSG0	Eif2s3y	chrY:34698	q1	q2	OK	0	13.9184
ENSMUSG0	ENSMUSG0	Ctxn3	chr18:5762	q1	q2	OK	0.188204	12.0089
ENSMUSG0	ENSMUSG0	Ampd1	chr3:10287	q1	q2	OK	1.88288	11.1504
ENSMUSG0	ENSMUSG0	Myo18b	chr5:11311	q1	q2	OK	0.261613	1.95885
ENSMUSG0	ENSMUSG0	Kbtbd5	chr9:12168	q1	q2	OK	0.165489	4.86699
ENSMUSG0	ENSMUSG0	Ces1h	chr8:95875	q1	q2	OK	0.382218	3.5584
ENSMUSG0	ENSMUSG0	Kbtbd10	chr2:69508	q1	q2	OK	1.84283	13.3581
ENSMUSG0	ENSMUSG0	Xirp1	chr9:11992	q1	q2	OK	0.288108	2.90119
ENSMUSG0	ENSMUSG0	Tmem182	chr1:40862	q1	q2	OK	0.696558	4.78939
ENSMUSG0	ENSMUSG0	1110002E2	chr3:13771	q1	q2	OK	0.143624	1.4391
ENSMUSG0	ENSMUSG0	Fitm1	chr14:5619	q1	q2	OK	1.24641	5.78979
ENSMUSG0	ENSMUSG0	Myoz2	chr3:12270	q1	q2	OK	9.03548	28.4691

ENSMUSG0	ENSMUSG0	Asb16	chr11:1021	q1	q2	OK	0.135141	1.06097
ENSMUSG0	ENSMUSG0	Pthlh	chr6:14720	q1	q2	OK	2.2046	8.35695
ENSMUSG0	ENSMUSG0	Mmp12	chr9:73443	q1	q2	OK	66.3788	16.6419
ENSMUSG0	ENSMUSG0	Gfod1	chr13:4329	q1	q2	OK	4.1548	1.46493
ENSMUSG0	ENSMUSG0	Myh7	chr14:5558	q1	q2	OK	7.13514	55.7893
ENSMUSG0	ENSMUSG0	Lypd3	chr7:25421	q1	q2	OK	0.13237	1.84983
ENSMUSG0	ENSMUSG0	Colq	chr14:3233	q1	q2	OK	11.4136	4.24572
ENSMUSG0	ENSMUSG0	Eno3	chr11:7047	q1	q2	OK	31.2121	132.376
ENSMUSG0	ENSMUSG0	Lyz1	chr10:1167	q1	q2	OK	278.565	112.949
ENSMUSG0	ENSMUSG0	Iglc3	chr16:1906	q1	q2	OK	58.7099	178.857
ENSMUSG0	ENSMUSG0	Slc12a2	chr18:5803	q1	q2	OK	27.6048	48.4922
ENSMUSG0	ENSMUSG0	Ddah1	chr3:14542	q1	q2	OK	13.5503	5.16771
ENSMUSG0	ENSMUSG0	Tspan11	chr6:12783	q1	q2	OK	21.6454	48.9727
ENSMUSG0	ENSMUSG0	1110059M:	chrX:42444	q1	q2	OK	0.141204	4.04844
ENSMUSG0	ENSMUSG0	Ppp1r16b	chr2:15849	q1	q2	OK	17.2299	8.31013
ENSMUSG0	ENSMUSG0	Alpk3	chr7:88202	q1	q2	OK	0.407484	1.48504
ENSMUSG0	ENSMUSG0	Pnpla3	chr15:8399	q1	q2	OK	3.17051	0.801452
ENSMUSG0	ENSMUSG0	Cxcr5	chr9:44319	q1	q2	OK	8.57986	2.48778
ENSMUSG0	ENSMUSG0	Gbp10	chr5:10564	q1	q2	OK	1.18329	0.209695
ENSMUSG0	ENSMUSG0	Tspan7	chrX:10062	q1	q2	OK	330.715	143.903
ENSMUSG0	ENSMUSG0	Actg2	chr6:83462	q1	q2	OK	11.6264	28.6839
ENSMUSG0	ENSMUSG0	Cav3	chr6:11240	q1	q2	OK	3.41164	11.5866
ENSMUSG0	ENSMUSG0	Apol11b	chr15:7746	q1	q2	OK	0.898622	4.8239
ENSMUSG0	ENSMUSG0	Tmod4	chr3:94928	q1	q2	OK	1.26386	7.05122
ENSMUSG0	ENSMUSG0	Trdn	chr10:3280	q1	q2	OK	2.18045	12.9345
ENSMUSG0	ENSMUSG0	Pcdh12	chr18:3842	q1	q2	OK	7.19698	2.84723
ENSMUSG0	ENSMUSG0	Maoa	chrX:16196	q1	q2	OK	14.3057	6.5584
ENSMUSG0	ENSMUSG0	Tspan18	chr2:93041	q1	q2	OK	20.6057	8.82024
ENSMUSG0	ENSMUSG0	Pcsk2	chr2:14337	q1	q2	OK	0.0332372	0.571645
ENSMUSG0	ENSMUSG0	Stac3	chr10:1269	q1	q2	OK	0.863008	6.69729
ENSMUSG0	ENSMUSG0	Abcb1a	chr5:86600	q1	q2	OK	8.52425	3.30974
ENSMUSG0	ENSMUSG0	Gjb3	chr4:12700	q1	q2	OK	4.04234	11.1883
ENSMUSG0	ENSMUSG0	Lce3c	chr3:92748	q1	q2	OK	0	0.831223
ENSMUSG0	ENSMUSG0	Obscn	chr11:5880	q1	q2	OK	2.31434	8.7079
ENSMUSG0	ENSMUSG0	Mcc	chr18:4458	q1	q2	OK	8.94364	4.37352
ENSMUSG0	ENSMUSG0	Cox4i2	chr2:15257	q1	q2	OK	91.7688	40.0449

ENSMUSG0	ENSMUSG0	Wnt9b	chr11:1035 q1	q2	OK	0.143535	0.840396
ENSMUSG0	ENSMUSG0	Tspan13	chr12:3674 q1	q2	OK	149.934	74.3671
ENSMUSG0	ENSMUSG0	Wnt10a	chr1:74838 q1	q2	OK	0.163287	1.2145
ENSMUSG0	ENSMUSG0	Yipf7	chr5:69907 q1	q2	OK	1.06064	4.8241
ENSMUSG0	ENSMUSG0	Foxf2	chr13:3171 q1	q2	OK	14.9181	5.73714
ENSMUSG0	ENSMUSG0	Faim3	chr1:13276 q1	q2	OK	13.9533	3.36506
ENSMUSG0	ENSMUSG0	Otop1	chr5:38667 q1	q2	OK	1.7091	0.094953
ENSMUSG0	ENSMUSG0	Kcnf1	chr12:1717 q1	q2	OK	0.817453	2.61335
ENSMUSG0	ENSMUSG0	Nipal1	chr5:73039 q1	q2	OK	1.27998	3.57728
ENSMUSG0	ENSMUSG0	Hkdc1	chr10:6184 q1	q2	OK	3.9273	8.86814
ENSMUSG0	ENSMUSG0	Tpm2	chr4:43527 q1	q2	OK	45.7824	235.139
ENSMUSG0	ENSMUSG0	Kel	chr6:41636 q1	q2	OK	0.0765881	0.874683
ENSMUSG0	ENSMUSG0	Klk14	chr7:50945 q1	q2	OK	4.01524	11.9138
ENSMUSG0	ENSMUSG0	Rpsa-ps10	chr3:14973 q1	q2	OK	31.0339	86.7324
ENSMUSG0	ENSMUSG0	Dsc3	chr18:2011 q1	q2	OK	0.0248513	0.761704
ENSMUSG0	ENSMUSG0	2310042D1	chr4:15559 q1	q2	OK	1.61664	4.29031
ENSMUSG0	ENSMUSG0	Rbfox1	chr16:6809 q1	q2	OK	0.31052	1.29956
ENSMUSG0	ENSMUSG0	Hsd11b1	chr1:19504 q1	q2	OK	104.94	50.7378
ENSMUSG0	ENSMUSG0	Celf4	chr18:2563 q1	q2	OK	1.79403	4.48358
ENSMUSG0	ENSMUSG0	Pla2g1b	chr5:11591 q1	q2	OK	20.9543	55.3058
ENSMUSG0	ENSMUSG0	Il2rg	chrX:98456 q1	q2	OK	51.1988	22.6093
ENSMUSG0	ENSMUSG0	Azgp1	chr5:13842 q1	q2	OK	1.5966	7.40231
ENSMUSG0	ENSMUSG0	Mfap4	chr11:6129 q1	q2	OK	505.845	187.95
ENSMUSG0	ENSMUSG0	Lipg	chr18:7509 q1	q2	OK	6.79374	2.2921
ENSMUSG0	ENSMUSG0	Sema3e	chr5:14025 q1	q2	OK	14.9955	7.35739
ENSMUSG0	ENSMUSG0	Ecscr	chr18:3587 q1	q2	OK	87.1359	40.6808
ENSMUSG0	ENSMUSG0	Bmp4	chr14:4699 q1	q2	OK	32.5808	72.7822
ENSMUSG0	ENSMUSG0	Adcyap1	chr17:9359 q1	q2	OK	0.219265	1.49377
ENSMUSG0	ENSMUSG0	Camk2a	chr18:6108 q1	q2	OK	0.603508	2.67098
ENSMUSG0	ENSMUSG0	Gbp2	chr3:14228 q1	q2	OK	37.353	17.1255
ENSMUSG0	ENSMUSG0	Dgat2	chr7:10630 q1	q2	OK	20.8581	9.20944
ENSMUSG0	ENSMUSG0	Jph1	chr1:16987 q1	q2	OK	0.19474	1.51232
ENSMUSG0	ENSMUSG0	Cd109	chr9:78463 q1	q2	OK	0.237211	0.893896
ENSMUSG0	ENSMUSG0	H2-T24	chr17:3614 q1	q2	OK	22.2796	9.35865
ENSMUSG0	ENSMUSG0	Mb	chr15:7684 q1	q2	OK	94.2955	530.962
ENSMUSG0	ENSMUSG0	Gnb3	chr6:12478 q1	q2	OK	2.91128	0.264996

ENSMUSG0	ENSMUSG0	Zfp385b	chr2:77248	q1	q2	OK	2.20845	5.73965
ENSMUSG0	ENSMUSG0	1810046K0	chr9:51097	q1	q2	OK	3.92555	0.5099
ENSMUSG0	ENSMUSG0	Krt80	chr15:1011	q1	q2	OK	61.0821	28.3029
ENSMUSG0	ENSMUSG0	Ceacam10	chr7:25562	q1	q2	OK	8.62709	1.57977
ENSMUSG0	ENSMUSG0	Cav2	chr6:17231	q1	q2	OK	123.528	59.5073
ENSMUSG0	ENSMUSG0	Hpx	chr7:11274	q1	q2	OK	2.83314	8.43395
ENSMUSG0	ENSMUSG0	2310046A0	chr9:76949	q1	q2	OK	1.51822	5.64668
ENSMUSG0	ENSMUSG0	Bcl2l15	chr3:10363	q1	q2	OK	1.64431	6.79881
ENSMUSG0	ENSMUSG0	Lrtm1	chr14:2971	q1	q2	OK	0.0446922	0.969069
ENSMUSG0	ENSMUSG0	Myh13	chr11:6713	q1	q2	OK	0.297702	0.992924
ENSMUSG0	ENSMUSG0	Sfta2	chr17:3573	q1	q2	OK	213.024	487.056
ENSMUSG0	ENSMUSG0	Lrrc32	chr7:10564	q1	q2	OK	19.4715	9.4162
ENSMUSG0	ENSMUSG0	Gdpd2	chrX:97925	q1	q2	OK	7.67761	15.9227
ENSMUSG0	ENSMUSG0	Wscd1	chr11:7156	q1	q2	OK	13.2431	5.54616
ENSMUSG0	ENSMUSG0	Gpd1	chr15:9954	q1	q2	OK	70.6259	33.6149
ENSMUSG0	ENSMUSG0	Atp13a4	chr16:2939	q1	q2	OK	3.35996	7.74455
ENSMUSG0	ENSMUSG0	E130012A1	chr11:9748	q1	q2	OK	4.87893	11.5077
ENSMUSG0	ENSMUSG0	Tubb4a	chr17:5721	q1	q2	OK	8.67797	2.96517
ENSMUSG0	ENSMUSG0	Capn3	chr2:12028	q1	q2	OK	0.857526	4.00798
ENSMUSG0	ENSMUSG0	Sdc3	chr4:13034	q1	q2	OK	28.6133	14.0775
ENSMUSG0	ENSMUSG0	5730559C1	chr1:13811	q1	q2	OK	2.76039	7.33893
ENSMUSG0	ENSMUSG0	Lad1	chr1:13771	q1	q2	OK	5.44952	11.4827
ENSMUSG0	ENSMUSG0	Rhbdl2	chr4:12346	q1	q2	OK	1.02846	3.7992
ENSMUSG0	ENSMUSG0	Gjb1	chrX:98571	q1	q2	OK	7.68567	18.9206
ENSMUSG0	ENSMUSG0	Gja4	chr4:12698	q1	q2	OK	49.9462	24.571
ENSMUSG0	ENSMUSG0	9330188P0	chr14:1059	q1	q2	OK	0.389523	2.60739
ENSMUSG0	ENSMUSG0	Aldh1a2	chr9:71063	q1	q2	OK	18.828	8.52459
ENSMUSG0	ENSMUSG0	Plscr2	chr9:92170	q1	q2	OK	31.7127	14.1624
ENSMUSG0	ENSMUSG0	Chst2	chr9:95301	q1	q2	OK	4.337	1.80787
ENSMUSG0	ENSMUSG0	Tnfsf10	chr3:27215	q1	q2	OK	35.3219	17.5483
ENSMUSG0	ENSMUSG0	Nkain4	chr2:18066	q1	q2	OK	54.4853	22.7715
ENSMUSG0	ENSMUSG0	Rac2	chr15:7838	q1	q2	OK	40.0306	18.761
ENSMUSG0	ENSMUSG0	Msln	chr17:2588	q1	q2	OK	72.4955	33.7231
ENSMUSG0	ENSMUSG0	Slc8a3	chr12:8229	q1	q2	OK	0.0762287	0.414051
ENSMUSG0	ENSMUSG0	Cdh5	chr8:10662	q1	q2	OK	131.755	59.9344
ENSMUSG0	ENSMUSG0	Apoa1	chr9:46036	q1	q2	OK	0.313573	2.39181

ENSMUSG0	ENSMUSG0	Pkhd1	chr1:20047	q1	q2	OK	0.0901741	0.341964
ENSMUSG0	ENSMUSG0	Crct1	chr3:92818	q1	q2	OK	0.518086	3.68096
ENSMUSG0	ENSMUSG0	Gdf3	chr6:12255	q1	q2	OK	0.166639	1.01493
ENSMUSG0	ENSMUSG0	Magohb	chr6:13123	q1	q2	OK	12.7015	31.4297
ENSMUSG0	ENSMUSG0	Gm98	chr19:1028	q1	q2	OK	13.0234	6.28341
ENSMUSG0	ENSMUSG0	Zmat4	chr8:24746	q1	q2	OK	0.267703	1.24936
ENSMUSG0	ENSMUSG0	Cldn5	chr16:1877	q1	q2	OK	570.593	259.611
ENSMUSG0	ENSMUSG0	Sdpr	chr1:51345	q1	q2	OK	199.017	90.1859
ENSMUSG0	ENSMUSG0	Ntm	chr9:28802	q1	q2	OK	0.208317	0.871266
ENSMUSG0	ENSMUSG0	C2cd4b	chr9:67606	q1	q2	OK	7.66618	19.6433
ENSMUSG0	ENSMUSG0	Coro6	chr11:7727	q1	q2	OK	4.35692	10.4063
ENSMUSG0	ENSMUSG0	Cd79b	chr11:1061	q1	q2	OK	67.8789	31.2605
ENSMUSG0	ENSMUSG0	Tnfrsf13c	chr15:8205	q1	q2	OK	6.81096	1.73427
ENSMUSG0	ENSMUSG0	Krt23	chr11:9933	q1	q2	OK	18.7189	37.9611
ENSMUSG0	ENSMUSG0	Adcy8	chr15:6453	q1	q2	OK	3.44381	1.19
ENSMUSG0	ENSMUSG0	Slc15a1	chr14:1218	q1	q2	OK	0.950545	2.69098
ENSMUSG0	ENSMUSG0	Fmo4	chr1:16472	q1	q2	OK	1.50389	4.85236
ENSMUSG0	ENSMUSG0	Npy	chr6:49772	q1	q2	OK	7.68384	0.518835
ENSMUSG0	ENSMUSG0	Myl12b	chr17:7132	q1	q2	OK	345.691	172.792
ENSMUSG0	ENSMUSG0	Apoc1	chr7:20274	q1	q2	OK	110.277	233.012
ENSMUSG0	ENSMUSG0	Ccrl2	chr9:11095	q1	q2	OK	12.6462	4.94548
ENSMUSG0	ENSMUSG0	Pvalb	chr15:7802	q1	q2	OK	3.91624	14.9961
ENSMUSG0	ENSMUSG0	Spp1	chr5:10486	q1	q2	OK	309.816	141.921
ENSMUSG0	ENSMUSG0	Abcc9	chr6:14253	q1	q2	OK	1.23363	2.82295
ENSMUSG0	ENSMUSG0	Cntnap2	chr6:45010	q1	q2	OK	0.515934	4.27716
ENSMUSG0	ENSMUSG0	Ndst1	chr18:6084	q1	q2	OK	36.3747	17.6996
ENSMUSG0	ENSMUSG0	Slfn5	chr11:8276	q1	q2	OK	8.5525	3.70376
ENSMUSG0	ENSMUSG0	Pdzd2	chr15:1228	q1	q2	OK	17.2813	7.92657
ENSMUSG0	ENSMUSG0	Bank1	chr3:13571	q1	q2	OK	8.64224	2.15676
ENSMUSG0	ENSMUSG0	Ttn	chr2:76542	q1	q2	OK	0.512546	3.61612
ENSMUSG0	ENSMUSG0	AI427809	chr4:53274	q1	q2	OK	6.27764	0.946148
ENSMUSG0	ENSMUSG0	Cyp2a4	chr7:27092	q1	q2	OK	0.430749	1.82353

log2.fold_change.	test_stat	p_value	q_value	significant
-1.4119	-2.65778	5.00E-05	0.0042944	yes
3.42022	4.33541	5.00E-05	0.0042944	yes
-1.44095	-2.54444	5.00E-05	0.0042944	yes
1.54984	3.1239	5.00E-05	0.0042944	yes
1.94832	2.76107	5.00E-05	0.0042944	yes
1.74782	2.7477	5.00E-05	0.0042944	yes
3.03354	3.03209	5.00E-05	0.0042944	yes
-1.99912	-3.26889	5.00E-05	0.0042944	yes
2.97126	2.58848	5.00E-05	0.0042944	yes
-1.27903	-2.53507	5.00E-05	0.0042944	yes
Inf	NA	5.00E-05	0.0042944	yes
1.60788	2.87187	5.00E-05	0.0042944	yes
4.70638	5.32443	5.00E-05	0.0042944	yes
2.2138	3.04683	5.00E-05	0.0042944	yes
3.75577	4.48046	5.00E-05	0.0042944	yes
2.70312	4.83668	5.00E-05	0.0042944	yes
-1.75488	-2.56261	5.00E-05	0.0042944	yes
3.67809	3.31803	5.00E-05	0.0042944	yes
1.69455	3.95457	5.00E-05	0.0042944	yes
-1.31135	-2.61027	5.00E-05	0.0042944	yes
1.97379	2.61095	5.00E-05	0.0042944	yes
1.4616	2.12948	5.00E-05	0.0042944	yes
1.86414	2.77951	5.00E-05	0.0042944	yes
2.70759	3.87579	5.00E-05	0.0042944	yes
2.39894	3.34521	5.00E-05	0.0042944	yes
5.38708	3.20267	5.00E-05	0.0042944	yes
2.8405	3.45574	5.00E-05	0.0042944	yes
5.1089	5.62699	5.00E-05	0.0042944	yes
1.87264	3.93505	5.00E-05	0.0042944	yes
-1.50033	-2.69663	5.00E-05	0.0042944	yes
3.08878	3.08804	5.00E-05	0.0042944	yes
2.39227	2.96265	5.00E-05	0.0042944	yes
1.40771	2.44244	5.00E-05	0.0042944	yes
2.56189	2.65398	5.00E-05	0.0042944	yes
1.7555	2.66191	5.00E-05	0.0042944	yes

4.33464	4.17035	5.00E-05	0.0042944	yes
3.06738	3.13916	5.00E-05	0.0042944	yes
1.63302	2.89969	5.00E-05	0.0042944	yes
2.15465	3.34028	5.00E-05	0.0042944	yes
1.34203	2.30868	5.00E-05	0.0042944	yes
5.93465	5.46188	5.00E-05	0.0042944	yes
-1.88617	-3.36547	5.00E-05	0.0042944	yes
-2.51772	-3.63885	5.00E-05	0.0042944	yes
2.14968	2.67488	5.00E-05	0.0042944	yes
1.5266	2.41456	5.00E-05	0.0042944	yes
6.05813	4.44632	5.00E-05	0.0042944	yes
2.99012	3.0458	5.00E-05	0.0042944	yes
5.4706	4.33291	5.00E-05	0.0042944	yes
3.86026	3.31612	5.00E-05	0.0042944	yes
1.33257	2.52581	5.00E-05	0.0042944	yes
2.29567	3.6357	5.00E-05	0.0042944	yes
4.579	4.62437	5.00E-05	0.0042944	yes
-2.27168	-3.86875	5.00E-05	0.0042944	yes
-1.57799	-3.2568	5.00E-05	0.0042944	yes
5.13303	5.31309	5.00E-05	0.0042944	yes
3.10586	2.27388	5.00E-05	0.0042944	yes
-1.25752	-2.57453	5.00E-05	0.0042944	yes
2.52564	2.63644	5.00E-05	0.0042944	yes
-2.15878	-3.00099	5.00E-05	0.0042944	yes
2.86692	3.02154	5.00E-05	0.0042944	yes
1.95431	2.47824	5.00E-05	0.0042944	yes
3.19291	2.9661	5.00E-05	0.0042944	yes
4.45822	3.44996	5.00E-05	0.0042944	yes
-1.32801	-2.47401	5.00E-05	0.0042944	yes
-1.6932	-2.65132	5.00E-05	0.0042944	yes
3.97291	3.3946	5.00E-05	0.0042944	yes
3.60292	4.85264	5.00E-05	0.0042944	yes
-2.08252	-2.79248	5.00E-05	0.0042944	yes
1.4523	3.93927	5.00E-05	0.0042944	yes
2.34081	3.37339	5.00E-05	0.0042944	yes
2.3316	3.49863	5.00E-05	0.0042944	yes

1.96673	2.4513	5.00E-05	0.0042944	yes
1.52608	2.42889	5.00E-05	0.0042944	yes
4.8509	8.12163	5.00E-05	0.0042944	yes
4.62932	4.31299	5.00E-05	0.0042944	yes
3.99029	4.77239	5.00E-05	0.0042944	yes
2.61487	3.77168	5.00E-05	0.0042944	yes
-2.34326	-3.65558	5.00E-05	0.0042944	yes
3.49999	4.4228	5.00E-05	0.0042944	yes
6.06285	3.40793	5.00E-05	0.0042944	yes
1.64672	3.15727	5.00E-05	0.0042944	yes
5.41221	4.97697	5.00E-05	0.0042944	yes
2.78823	4.26623	5.00E-05	0.0042944	yes
2.13608	2.61847	5.00E-05	0.0042944	yes
1.96099	3.13252	5.00E-05	0.0042944	yes
-1.22058	-2.52302	5.00E-05	0.0042944	yes
5.35632	6.49734	5.00E-05	0.0042944	yes
1.46094	2.93893	5.00E-05	0.0042944	yes
-7.46458	-8.11232	5.00E-05	0.0042944	yes
2.2828	6.39676	5.00E-05	0.0042944	yes
1.45393	2.98811	5.00E-05	0.0042944	yes
1.86937	3.18745	5.00E-05	0.0042944	yes
1.81119	2.62741	5.00E-05	0.0042944	yes
3.36266	2.73666	5.00E-05	0.0042944	yes
1.69215	2.36146	5.00E-05	0.0042944	yes
2.20326	3.03476	5.00E-05	0.0042944	yes
-1.47975	-2.99998	5.00E-05	0.0042944	yes
1.50861	3.14594	5.00E-05	0.0042944	yes
2.88396	3.33674	5.00E-05	0.0042944	yes
3.46294	3.36611	5.00E-05	0.0042944	yes
1.82966	3.09452	5.00E-05	0.0042944	yes
-1.59044	-3.15886	5.00E-05	0.0042944	yes
1.94353	2.82823	5.00E-05	0.0042944	yes
-1.24965	-2.27791	5.00E-05	0.0042944	yes
-2.06545	-2.28727	5.00E-05	0.0042944	yes
1.63967	2.39098	5.00E-05	0.0042944	yes
2.37332	3.44076	5.00E-05	0.0042944	yes

-1.16552	-2.3251	5.00E-05	0.0042944	yes
2.86113	2.92721	5.00E-05	0.0042944	yes
3.04264	4.02809	5.00E-05	0.0042944	yes
2.3806	3.16352	5.00E-05	0.0042944	yes
2.31447	3.28938	5.00E-05	0.0042944	yes
-2.80801	-5.83613	5.00E-05	0.0042944	yes
4.12557	6.06536	5.00E-05	0.0042944	yes
-1.37799	-2.5903	5.00E-05	0.0042944	yes
-1.71909	-2.61616	5.00E-05	0.0042944	yes
1.24152	2.47274	5.00E-05	0.0042944	yes
2.86035	3.89301	5.00E-05	0.0042944	yes
-1.25419	-2.55217	5.00E-05	0.0042944	yes
Inf	NA	5.00E-05	0.0042944	yes
2.28254	5.56237	5.00E-05	0.0042944	yes
1.52706	2.58424	5.00E-05	0.0042944	yes
1.30535	2.41943	5.00E-05	0.0042944	yes
2.12033	2.92421	5.00E-05	0.0042944	yes
2.02508	4.23003	5.00E-05	0.0042944	yes
2.30812	3.09561	5.00E-05	0.0042944	yes
2.55266	3.33649	5.00E-05	0.0042944	yes
-1.58406	-3.20508	5.00E-05	0.0042944	yes
3.73891	3.65388	5.00E-05	0.0042944	yes
3.83527	7.27218	5.00E-05	0.0042944	yes
8.60561	9.05644	5.00E-05	0.0042944	yes
3.40521	4.03056	5.00E-05	0.0042944	yes
3.20709	3.98057	5.00E-05	0.0042944	yes
2.10201	2.60527	5.00E-05	0.0042944	yes
6.45245	7.59376	5.00E-05	0.0042944	yes
-2.33236	-3.64015	5.00E-05	0.0042944	yes
2.91723	4.09695	5.00E-05	0.0042944	yes
1.94001	2.28263	5.00E-05	0.0042944	yes
1.88491	3.15921	5.00E-05	0.0042944	yes
3.37727	3.69853	5.00E-05	0.0042944	yes
3.26589	2.73388	5.00E-05	0.0042944	yes
2.16752	2.77885	5.00E-05	0.0042944	yes
1.43577	2.45707	5.00E-05	0.0042944	yes

	-1.26452	-2.39362	5.00E-05	0.0042944	yes
	6.66819	5.75927	5.00E-05	0.0042944	yes
Inf	NA		5.00E-05	0.0042944	yes
	2.00867	3.62421	5.00E-05	0.0042944	yes
	2.88554	3.6165	5.00E-05	0.0042944	yes
	3.99885	4.59318	5.00E-05	0.0042944	yes
	1.8402	3.01508	5.00E-05	0.0042944	yes
	9.46495	6.86029	5.00E-05	0.0042944	yes
	6.00307	3.60635	5.00E-05	0.0042944	yes
	1.77439	2.64368	5.00E-05	0.0042944	yes
	-1.32681	-2.18922	5.00E-05	0.0042944	yes
Inf	NA		5.00E-05	0.0042944	yes
	7.61388	7.84533	5.00E-05	0.0042944	yes
	4.64331	6.9258	5.00E-05	0.0042944	yes
	2.27363	2.90274	5.00E-05	0.0042944	yes
	6.09083	6.04508	5.00E-05	0.0042944	yes
	3.36614	4.2661	5.00E-05	0.0042944	yes
	2.5852	3.05316	5.00E-05	0.0042944	yes
	1.54478	2.85712	5.00E-05	0.0042944	yes
	-1.2908	-2.60301	5.00E-05	0.0042944	yes
	1.57042	2.98713	5.00E-05	0.0042944	yes
	3.1826	2.61709	5.00E-05	0.0042944	yes
	5.87237	6.88091	5.00E-05	0.0042944	yes
	2.76473	4.09171	5.00E-05	0.0042944	yes
Inf	NA		5.00E-05	0.0042944	yes
	5.99567	6.5342	5.00E-05	0.0042944	yes
	2.56608	2.19638	5.00E-05	0.0042944	yes
	2.9045	3.53467	5.00E-05	0.0042944	yes
	4.87822	5.56226	5.00E-05	0.0042944	yes
	3.21876	2.65961	5.00E-05	0.0042944	yes
	2.85772	3.95939	5.00E-05	0.0042944	yes
	3.33196	4.04819	5.00E-05	0.0042944	yes
	2.78153	3.35604	5.00E-05	0.0042944	yes
	3.3248	3.83228	5.00E-05	0.0042944	yes
	2.21574	2.93363	1.00E-04	0.0080434	yes
	1.65573	3.13506	1.00E-04	0.0080434	yes

2.97284	2.76931	1.00E-04	0.0080434	yes
1.92246	2.9694	1.00E-04	0.0080434	yes
-1.9959	-2.61332	1.00E-04	0.0080434	yes
-1.50395	-2.45739	1.00E-04	0.0080434	yes
2.96698	2.80733	1.00E-04	0.0080434	yes
3.80475	3.34558	1.00E-04	0.0080434	yes
-1.42667	-2.28915	1.00E-04	0.0080434	yes
2.08447	2.40072	1.00E-04	0.0080434	yes
-1.30234	-2.85334	1.00E-04	0.0080434	yes
1.60713	3.582	1.00E-04	0.0080434	yes
1.14247	1.56989	0.00015	0.0112886	yes
-1.39073	-2.48921	0.00015	0.0112886	yes
1.17792	2.33317	0.00015	0.0112886	yes
4.84152	4.2377	0.00015	0.0112886	yes
-1.05197	-2.12949	0.00015	0.0112886	yes
1.86569	2.25809	0.00015	0.0112886	yes
-1.98403	-2.53425	0.00015	0.0112886	yes
-1.7861	-2.55863	0.00015	0.0112886	yes
-2.49643	-2.40527	0.00015	0.0112886	yes
-1.20049	-2.08976	0.00015	0.0112886	yes
1.30284	2.28713	0.00015	0.0112886	yes
1.76392	2.78941	0.00015	0.0112886	yes
2.42442	2.77542	0.00015	0.0112886	yes
2.48003	2.14552	2.00E-04	0.0142075	yes
2.56852	1.65183	2.00E-04	0.0142075	yes
-1.33783	-2.30455	2.00E-04	0.0142075	yes
-1.12518	-2.15616	2.00E-04	0.0142075	yes
-1.22415	-2.31339	2.00E-04	0.0142075	yes
4.10425	3.18368	2.00E-04	0.0142075	yes
2.95613	1.99815	2.00E-04	0.0142075	yes
-1.36486	-2.41326	2.00E-04	0.0142075	yes
1.46873	2.22321	2.00E-04	0.0142075	yes
Inf	NA	2.00E-04	0.0142075	yes
1.91172	2.21113	2.00E-04	0.0142075	yes
-1.03207	-2.06342	2.00E-04	0.0142075	yes
-1.19638	-2.25999	0.00025	0.0169665	yes

2.54967	2.41473	0.00025	0.0169665	yes
-1.01159	-1.95279	0.00025	0.0169665	yes
2.89489	2.68839	0.00025	0.0169665	yes
2.18533	2.76099	0.00025	0.0169665	yes
-1.37866	-2.72668	0.00025	0.0169665	yes
-2.0519	-2.90748	0.00025	0.0169665	yes
-4.16988	-3.51072	0.00025	0.0169665	yes
1.67669	2.28158	0.00025	0.0169665	yes
1.48274	2.14149	0.00025	0.0169665	yes
1.17509	2.17331	3.00E-04	0.0197429	yes
2.36065	2.03376	3.00E-04	0.0197429	yes
3.51357	2.75221	3.00E-04	0.0197429	yes
1.56908	2.6081	3.00E-04	0.0197429	yes
1.48272	2.69803	3.00E-04	0.0197429	yes
4.93783	3.12647	3.00E-04	0.0197429	yes
1.40808	2.12656	3.00E-04	0.0197429	yes
2.06527	2.14249	0.00035	0.0220776	yes
-1.04843	-2.05834	0.00035	0.0220776	yes
1.32145	2.0524	0.00035	0.0220776	yes
1.40018	2.18262	0.00035	0.0220776	yes
-1.17919	-2.09524	0.00035	0.0220776	yes
2.21297	2.64177	0.00035	0.0220776	yes
-1.42835	-2.54647	0.00035	0.0220776	yes
-1.56754	-2.51275	0.00035	0.0220776	yes
-1.02726	-2.09226	0.00035	0.0220776	yes
-1.09892	-2.07167	0.00035	0.0220776	yes
1.15956	2.2417	4.00E-04	0.0244209	yes
2.7682	2.60679	4.00E-04	0.0244209	yes
2.14592	1.96804	4.00E-04	0.0244209	yes
-1.12508	-2.35435	4.00E-04	0.0244209	yes
-1.17942	-2.36335	4.00E-04	0.0244209	yes
2.95714	2.75302	4.00E-04	0.0244209	yes
1.91394	2.1465	4.00E-04	0.0244209	yes
-1.25135	-2.11072	4.00E-04	0.0244209	yes
2.49335	3.29861	0.00045	0.0268271	yes
-3.45761	-3.21141	0.00045	0.0268271	yes

1.37793	2.03069	0.00045	0.0268271	yes
-2.94461	-3.08034	0.00045	0.0268271	yes
-1.1098	-2.03261	0.00045	0.0268271	yes
-2.44916	-2.94855	0.00045	0.0268271	yes
-1.0537	-1.96011	5.00E-04	0.0287917	yes
1.57381	2.33302	5.00E-04	0.0287917	yes
1.89502	2.00659	5.00E-04	0.0287917	yes
2.0478	1.99212	5.00E-04	0.0287917	yes
4.4385	3.09154	5.00E-04	0.0287917	yes
1.73781	1.99524	5.00E-04	0.0287917	yes
1.19307	2.0704	5.00E-04	0.0287917	yes
-1.04815	-2.14321	5.00E-04	0.0287917	yes
1.05235	1.95625	0.00055	0.0308528	yes
-1.25569	-2.15339	0.00055	0.0308528	yes
-1.07109	-2.09309	0.00055	0.0308528	yes
1.20474	1.92577	0.00055	0.0308528	yes
1.23796	2.63905	0.00055	0.0308528	yes
-1.54924	-2.47219	0.00055	0.0308528	yes
2.22462	2.11429	0.00055	0.0308528	yes
-1.0233	-2.00947	6.00E-04	0.0326925	yes
1.4107	2.13419	6.00E-04	0.0326925	yes
1.07526	2.13313	6.00E-04	0.0326925	yes
1.88521	2.04104	6.00E-04	0.0326925	yes
1.29972	2.27606	6.00E-04	0.0326925	yes
-1.02342	-2.42702	6.00E-04	0.0326925	yes
2.74283	2.84995	6.00E-04	0.0326925	yes
-1.14317	-2.18111	0.00065	0.0350401	yes
-1.16299	-1.92059	0.00065	0.0350401	yes
-1.26241	-2.08882	0.00065	0.0350401	yes
-1.00923	-1.85816	7.00E-04	0.0376021	yes
-1.25864	-2.36991	0.00075	0.0394516	yes
-1.09337	-2.07098	0.00075	0.0394516	yes
-1.10416	-2.19541	0.00075	0.0394516	yes
2.4414	1.9522	0.00075	0.0394516	yes
-1.1364	-2.11388	8.00E-04	0.041366	yes
2.93123	3.00305	8.00E-04	0.041366	yes

1.92306	1.9791	8.00E-04	0.041366	yes
2.82882	2.57737	0.00085	0.0426459	yes
2.60658	2.45721	0.00085	0.0426459	yes
1.30713	1.99973	0.00085	0.0426459	yes
-1.05149	-2.01616	0.00085	0.0426459	yes
2.22248	1.59767	0.00085	0.0426459	yes
-1.13611	-2.43615	0.00085	0.0426459	yes
-1.14192	-2.05003	0.00085	0.0426459	yes
2.06434	1.57491	0.00085	0.0426459	yes
1.35745	2.82926	0.00085	0.0426459	yes
1.25608	2.00612	9.00E-04	0.0445661	yes
-1.11862	-2.04787	9.00E-04	0.0445661	yes
-1.97353	-2.0511	9.00E-04	0.0445661	yes
1.02002	2.15831	0.00095	0.0457022	yes
-1.53304	-2.21774	0.00095	0.0457022	yes
1.5013	1.95585	0.00095	0.0457022	yes
1.68999	1.89346	0.00095	0.0457022	yes
-3.88848	-4.52757	0.00095	0.0457022	yes
-1.00045	-2.46134	0.00095	0.0457022	yes
1.07927	1.89427	0.00095	0.0457022	yes
-1.35452	-2.33768	0.00095	0.0457022	yes
1.93705	2.00593	0.001	0.047065	yes
-1.12632	-1.91555	0.001	0.047065	yes
1.19429	1.90749	0.001	0.047065	yes
3.0514	2.55513	0.001	0.047065	yes
-1.03921	-1.96471	0.001	0.047065	yes
-1.20735	-1.93238	0.001	0.047065	yes
-1.12444	-2.05431	0.00105	0.0486649	yes
-2.00254	-1.89209	0.00105	0.0486649	yes
2.81869	1.4937	0.00105	0.0486649	yes
-2.73008	-3.31892	0.00105	0.0486649	yes
2.08181	2.4265	0.00105	0.0486649	yes
